# Developing a PRogram to Educate and Sensitize Caregivers to Reduce the Inappropriate Prescription Burden in the Elderly with Alzheimer’s Disease (D-PRESCRIBE-AD): Trial protocol and rationale of an open-label pragmatic, prospective randomized controlled trial

**DOI:** 10.1371/journal.pone.0297562

**Published:** 2024-02-12

**Authors:** Sonal Singh, Noelle M. Cocoros, Xiaojuan Li, Kathleen M. Mazor, Mary T. Antonelli, Lauren Parlett, Mark Paullin, Thomas P. Harkins, Yunping Zhou, Paula A. Rochon, Richard Platt, Inna Dashevsky, Carly Massino, Cassandra Saphirak, Sybil L. Crawford, Jerry H. Gurwitz

**Affiliations:** 1 Department of Family Medicine and Community Health, Division of Health Systems Science, Umass Chan Medical School, Worcester, Massachusetts, United States of America; 2 Department of Population Medicine, Harvard Medical School, Harvard Pilgrim Health Care Institute, Boston, Massachusetts, United States of America; 3 Division of Health Systems Science, UMass Chan Medical School, Worcester, Massachusetts, United States of America; 4 Tan Chingfen Graduate School of Nursing, UMass Chan Medical School, Worcester, Massachusetts, United States of America; 5 Carelon Research, Wilmington, Delaware, United States of America; 6 Humana Healthcare Research, Inc., (Humana), Louisville, Kentucky, United States of America; 7 Women’s Age Lab and Women’s College Research Institute, Women’s College Hospital, Department of Medicine, University of Toronto, Toronto, Ontario, Canada; 8 Division of Health System Science, UMass Chan Medical School, Tan Chingfen Graduate School of Nursing, Worcester, Massachusetts, United States of America; 9 Division of Geriatric Medicine and Division of Health Systems Science, UMass Chan Medical School, Worcester, Massachusetts, United States of America; PLOS: Public Library of Science, UNITED KINGDOM

## Abstract

**Context:**

Potentially inappropriate prescribing of medications in older adults, particular those with dementia, can lead to adverse drug events including falls and fractures, worsening cognitive impairment, emergency department visits, and hospitalizations. Educational mailings from health plans to patients and their providers to encourage deprescribing conversations may represent an effective, low-cost, “light touch”, approach to reducing the burden of potentially inappropriate prescription use in older adults with dementia.

**Objectives:**

The objective of the **D**eveloping a **PR**ogram to **E**ducate and **S**ensitize **C**aregivers to **R**educe the **I**nappropriate Prescription **B**urden in **E**lderly with **A**lzheimer’s **D**isease (D-PRESCRIBE-AD) trial is to evaluate the effect of a health plan based multi-faceted educational outreach intervention to community dwelling patients with dementia who are currently prescribed sedative/hypnotics, antipsychotics, or strong anticholinergics.

**Methods:**

The D-PRESCRIBE-AD is an open-label pragmatic, prospective randomized controlled trial (RCT) comparing three arms: 1) educational mailing to both the health plan patient and their prescribing physician (patient plus physician arm, n = 4814); 2) educational mailing to prescribing physician only (physician only arm, n = 4814); and 3) usual care (n = 4814) among patients with dementia enrolled in two large United States based health plans. The primary outcome is the absence of any dispensing of the targeted potentially inappropriate prescription during the 6-month study observation period after a 3-month black out period following the mailing. Secondary outcomes include dose-reduction, polypharmacy, healthcare utilization, mortality and therapeutic switching within targeted drug classes.

**Conclusion:**

This large pragmatic RCT will contribute to the evidence base on promoting deprescribing of potentially inappropriate medications among older adults with dementia. If successful, such light touch, inexpensive and highly scalable interventions have the potential to reduce the burden of potentially inappropriate prescribing for patients with dementia.

ClinicalTrials.gov Identifier: NCT05147428.

## Introduction

Potentially inappropriate prescribing includes the use of medications that may no longer be necessary or may increase the risk of harm. Potentially inappropriate prescribing in older adults can lead to adverse drug events, falls and fractures [1], worsening cognitive impairment [2], emergency department visits, and hospitalizations. It can also increase overall symptom burden and affect quality of life. The use of sedative/hypnotics, antipsychotics, and strong anticholinergic agents poses particular risks for older adults and may be more prevalent among those living with Alzheimer’s disease and Alzheimer’s disease-related dementias (AD/ADRD) due to a higher prevalence of multimorbidity and associated polypharmacy [3–5]. According to the American Geriatrics Society’s 2023 updated AGS Beers criteria^©^ these medications are considered potentially inappropriate medication classes for older adults and are typically best avoided by older adults with AD/ADRD in most circumstances [6].

Our prior work has suggested that mailing educational materials about potentially inappropriate medications to persons living with AD/ADRD in the community may promote deprescribing conversations [7]. To our knowledge, there are no large-scale pragmatic randomized controlled trials (RCTs), which evaluate whether health plan-based educational mailings to patients reduce inappropriate prescribing among older adults with AD/ADRD. Different approaches to effecting deprescribing have been reported in the literature. RCTs that evaluated the effect of peer-comparison letters mailed to physicians by health plans in other study populations and involving other drug classes, such as controlled substances, have shown inconsistent results [8, 9]. Other RCTs that promoted deprescribing generally involved using educational materials along with direct interaction between patients, providers, and pharmacists [10], or more intense engagement with patients at clinic sites [11].

Educational mailings from health plans to patients/caregivers and their providers to encourage deprescribing conversations may represent an effective, low-cost, “light touch” approach to reducing the burden of potentially inappropriate prescription use in older adults with dementia. The objective of the Developing a PRogram to Educate and Sensitize Caregivers to Reduce the Inappropriate Prescription Burden in Elderly with Alzheimer’s Disease (D-PRESCRIBE-AD) trial is to evaluate the effect of a health plan-based educational outreach intervention to community dwelling patients with AD/ADRD who are currently prescribed sedative/hypnotics, antipsychotics, or strong anticholinergics. The protocol is outlined below.

## Methods

### Study design and setting

**Fig 1:**
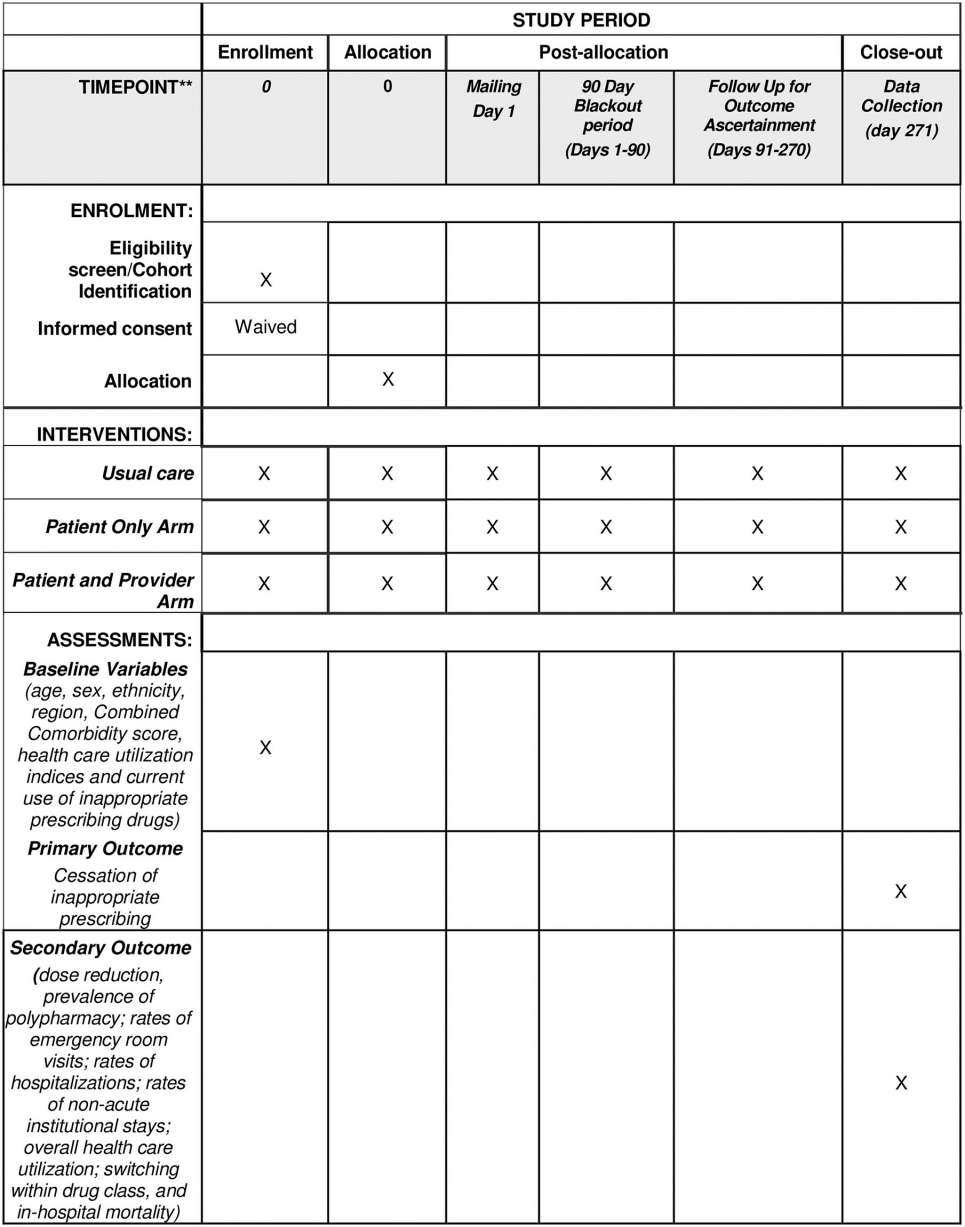
SPIRIT timeline for the D-PRESCRIBE AD RCT.

**Fig 2:**
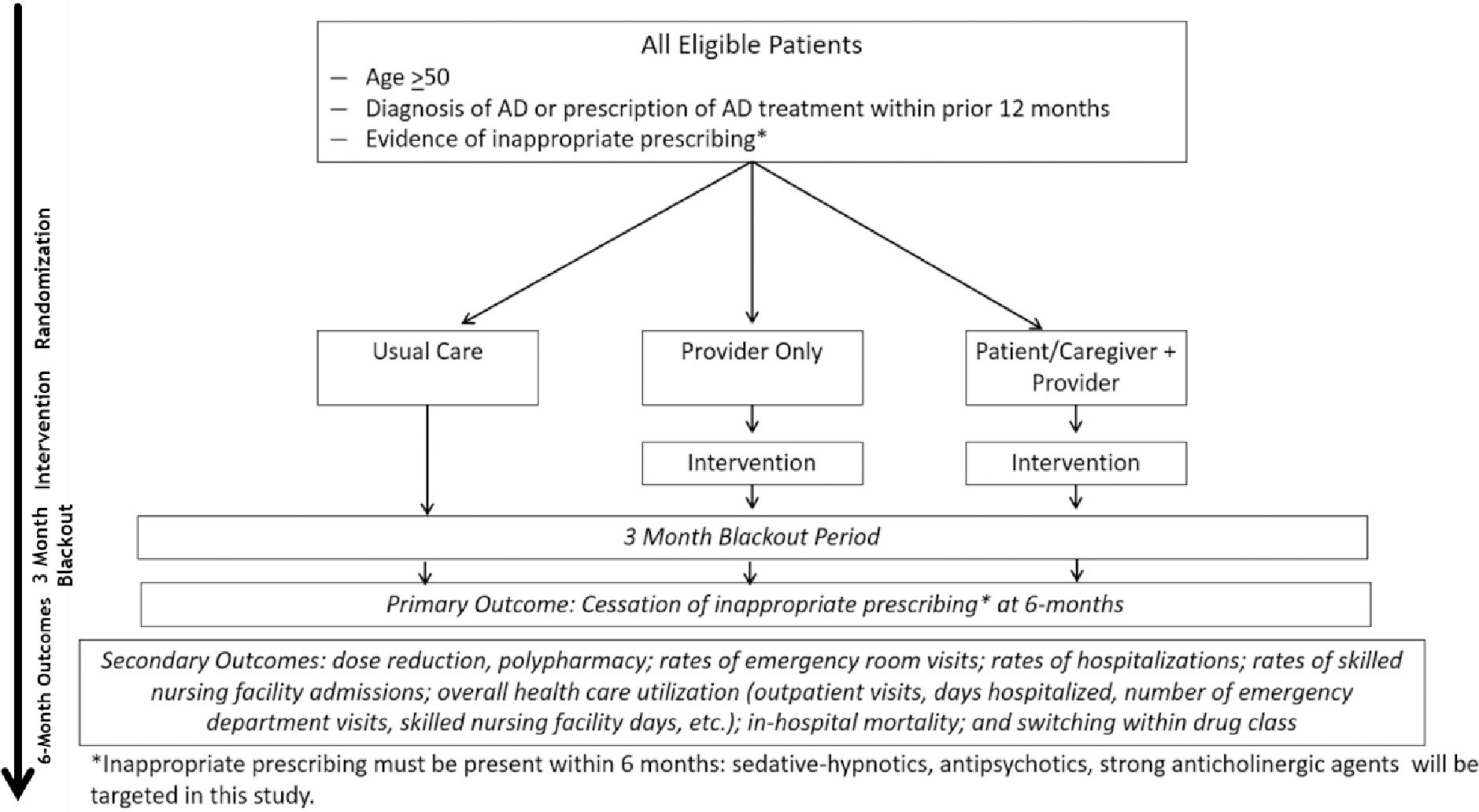
Trial design.

The SPIRIT timeline is shown in Fig 1. The D-PRESCRIBE-AD trial is a large health plan-based open-label pragmatic, prospective RCT comparing three arms: 1) educational mailing to both the health plan patient and their prescribing physician (patient plus physician arm, n = 4814); 2) educational mailing to prescribing physician only (physician only arm, n = 4814); and 3) usual care arm (n = 4814) as shown in Fig 2.

The study population includes patients enrolled in two United States (US) based health plans, Humana and Anthem, that participate in the NIH Collaboratory Distributed Research Network [12]. The domains of this pragmatic trial according to the pragmatic-explanatory continuum indicator summary (PRECIS) framework are shown in S1 Table.

[13, 14] This trial is registered at ClinicalTrials.gov Identifier: NCT05147428.

There was no recruitment for this pragmatic trial, but, we identified the cohort of participants on April 15, 2022 for health plan 1 and July 15, 2022 for health plan 2. At the time of protocol submission, we completed the mailing of the intervention to the three randomized arms. The collection of data, outcome ascertainment, and statistical analysis on primary and secondary outcomes is incomplete.

The NIH Pragmatic Trials Collaboratory Distributed Research Network leverages the US FDA’s Sentinel System’s technical infrastructure, which was originally developed for post-marketing medical product surveillance, but was also envisioned as a national resource, including one for pragmatic trials and other research studies [12, 15]. The health plans that participate in Sentinel transform their data into a common data model that harmonizes their data to streamline analyses and research across the health plans [16]. Those partners that are part of the Collaboratory are actively accruing data on ~75 million individuals with commercial insurance or Medicare Advantage coverage.

### Data and data management

Administrative claims data in the Sentinel System common data model from the two US based health plans, Humana and Anthem are being used for identification of cohort and measurement of outcomes in the study. These data used in this trial are accessed, maintained, and protected, as part of a “distributed network.” [16]. In a distributed network, data remain in their existing secure environments, rather than being consolidated into a single database. Health plans maintain physical and operational control over their electronic health data behind their institutional firewalls. A cohort identification analytic program written in SAS (Cary, NC), developed by the analytic coordinating center at Harvard Pilgrim Health Care Institute, was executed by the participating health plans separately to identify active patients who met eligibility criteria for the trial.

The statistical analysis for primary and secondary outcomes will be conducted on site at UMass Chan Medical School using deidentified individual-level data shared by the two health plans via a secure portal. A Data Use Agreement between the health plans and UMass Chan Medical School will facilitate data transfer and statistical analysis.

### Eligibility criteria for patients

Patients with continuous medical and pharmacy insurance coverage for at least one prior year were included if they met all of the following inclusion criteria: a) having AD/ADRD based on either presence of two ICD-10 diagnosis codes or two dispensings for a pharmacologic therapy used for AD (e.g., donepezil, rivastigmine, galantamine, or memantine) in the 365 days prior to or on cohort entry date; b) age ≥50 years of age as of cohort entry date; and c) evidence of potentially inappropriate prescribing of any of the three targeted drug classes (sedative hypnotics, antipsychotics, or strong anticholinergics) in the 3 months prior to or on the cohort entry date. The medications being targeted for deprescribing are listed in S2 Table. **Targeted Prescription Drugs**.

We excluded patients who reside in a nursing home or skilled nursing facility, receive palliative care, have incomplete or missing information on prescribing provider, or incomplete contact information for either patient or their prescribing provider. We also excluded patients who do not wish to be contacted by the health plan for research purposes. We also excluded patients who participated in the pilot mailing to test the feasibility of our approach.

### Eligibility criteria for providers

We included providers who were identified as the prescriber of the most recent dispensing of a target drug for an eligible patient. To address within-provider contamination only one randomly selected eligible patient was randomized and included in the study per prescribing provider who were associated with dispensing to multiple eligible patients.

### Allocation and randomization

A programmer at each health plan used locally stored patient ID numbers to identify the names and mailing addresses of eligible patients and utilized the locally stored provider ID numbers to identify the names and contact information of the prescribing provider. The health plans used these lists to implement mailings of the intervention materials. Patients were randomly assigned to one of the three treatment arms via random number generation in SAS using a uniform distribution. Randomization without replacement occurred at the individual patient level.

### Intervention development and stakeholder engagement

We adapted educational materials developed by Tannenbaum and colleagues to educational materials more specifically relevant to patients with AD/ADRD and their caregivers [17, 18]. The materials were informed by conversations with patients and caregivers, which suggested that educational mailings to the patient and caregiver would potentially prompt them to have discussions about deprescribing with their providers [7, 19]. In addition, we mailed the educational materials to 200 eligible patients with dementia from each of the two health plans. We solicited feedback regarding the acceptability of these materials. We received 34 responses which reinforced the findings of our qualitative study that these materials were acceptable to patients and caregivers [7], with several patients suggesting that they would bring this to their next medical appointment. These intervention may facilitate better outcomes for patients by developing their communication skills, improving their knowledge, and increasing their confidence to make healthcare decisions [20].

We developed separate sets of educational materials for antipsychotics, sedative-hypnotics, and strong anticholinergic agents. Patients who are on more than one class of potentially inappropriate medications only received educational materials relevant to one class of medications. For these patients we prioritized antipsychotic use, followed by sedatives/hypnotic use, followed by strong anticholinergic agents use.

We used an iterative process to adapt patient education materials known to be effective in reducing the use of inappropriate medications in older adults [17, 18]. Drafts of the materials were reviewed by stakeholders including patients with AD/ADRD, their caregivers, and providers in semi-structured interviews. We revised the materials based on stakeholder feedback in an iterative process, which were further reviewed by an advisory committee comprised of health plan leaders, geriatricians, and caregivers of patients with AD/ADRD. Finally, each participating health plan reviewed the materials to ensure they were consistent with other health plan messaging.

All mailings included a link to a study website (www.knowmymeds.org) for patients, caregivers, and healthcare providers which provided online access to all mailed materials.

The specific components of the educational materials to patients and providers, and the key concept conveyed in each, are listed in Tables 1 and 2. The Sample Patient Cover Letter is shown in S1 Appendix. The Fact Sheets are shown in S2 Appendix. The Pocket Cards are shown in S3 Appendix. The Sample Provider Cover Letter is shown in S4 Appendix. The Deprescribing Algorithms are shown in S5 Appendix. The Tapering Guides is shown in S6 Appendix.

**Table 1:** Patient/caregiver mailing enclosures.

Component	Key Concepts
Cover Letter (S1 Appendix)	Refers to specific drug prescribed to patient. Alert that drug may be inappropriate. Recommendation to share with caregivers. Recommendation to talk to provider about medication. Warning to NOT stop drug prematurely without talking to provider first
Information Sheet (S2 Appendix)	Focuses on drug class (includes specific drug prescribed to patient) List of potential side effects Recommendation to bring materials to appointment and discuss with provider
Pocket Card (S3 Appendix)	3 key questions to ask doctor Warning to NOT change any medications without consulting with provider first

**Table 2:** Provider mailing enclosures.

Component	Key Concepts
Cover Letter (S4 Appendix)	Includes patient specific information (name, dob, medication, medication initiation date) Alert that drug may be inappropriate for patient. Reference to materials enclosed
Deprescribing Algorithm (S5 Appendix)	Decision aid for specific drug class as to whether or not to recommend deprescribing (included for sedative-hypnotics, antipsychotics, and urinary anticholinergic drugs only)
Tapering Guide (S6 Appendix)	Detailed fillable tapering plan to share with patients and caregivers to assist with tapering of medication (included for sedative-hypnotics and antipsychotics only)
Patient Information Sheet and Pocket Card	See Table 1 for details on patient/caregiver materials

We mailed educational materials for both patients and physicians in the *patient plus physician arm*. Physicians received a copy of educational materials that were mailed to their patients, along with specific materials for physicians in the *physician only arm*. There were no educational materials mailed to patients in the *physician only arm*. There were no educational materials mailed to patients or physicians in the *usual care arm*.

### Study outcomes

#### Primary outcome

The primary outcome is defined as the absence of any dispensing of the targeted potentially inappropriate prescription during the 6-month study observation period after a 3-month black out period following the mailing. The blackout period was stipulated to allow an opportunity for the patient to contact and/or schedule a visit with their provider to discuss the use of the potentially inappropriate medication. Any prescriptions dispensed during the 3-month blackout period after the mailing will not be counted towards measurement of the primary outcome.

#### Secondary outcomes

All secondary outcomes will be assessed during the 6-month study observation period, after a blackout period of 3 months, subsequent to the mailing.

1. Dose reduction. Dose reduction will be measured as the proportion of patients who achieved a reduction in mean daily dose of 50% or more during the study observation period of 6 months compared to the mean daily dose in 6 months immediately prior to the mailing of educational materials.
2. Prevalence of polypharmacy. Polypharmacy will be defined as having dispensing of five or more different prescriptions [21]. We will compare the prevalence of polypharmacy between the treatment arms patients during the study observation period. We allowed the inclusion of participants with polypharmacy at baseline.
3. Healthcare utilization. We will also measure rates of hospitalizations, rates of emergency department visits, rates of non-acute institutional stays (e.g., skilled nursing facilities), and overall healthcare utilization(number of outpatient visits, days hospitalized, emergency department visits, and non-acute institutional days).
4. Death. We also intend to evaluate all-cause mortality as a secondary outcome. Out of hospital deaths cannot be reliably captured in available data.
5. Therapeutic switch. We will also evaluate therapeutic switches among study subjects who discontinue the targeted medication or who experience a dose reduction. We will determine if another agent within the targeted class was dispensed, e.g., for sedative/hypnotics the dispensing of an alternative agent within the class of sedative/hypnotics.

### Statistical analysis

#### Primary outcome

The three study arms will be compared using survival analyses regarding time to first dispensing of the targeted inappropriate prescription of their initial drug class within the 6-month study observation period. These analytic methods account for censoring due to death or disenrollment in the observation period.

We will first calculate crude arm-specific percentages, as well as Kaplan-Meier curves and log-rank testing, censoring at death or disenrollment from the health plan. The index date for the survival analysis will be Day 91 after the mailing.

Covariate-adjusted comparisons of the three arms will be estimated using Cox proportional hazards model. Relevant covariates include presence and timing of dispensing of the same potentially inappropriate medication class during the blackout period, as this may affect subsequent dispensing and thus the primary outcome. Comparing each active intervention arm to usual care, we hypothesize a hazard ratio of less than 1, indicating lower risk of having an inappropriate prescription in the 6-month study observation period in the active intervention arms. We also will conduct competing risk analyses [22] as well as cause-specific hazards modeling [23], to account for mortality, anticipated to be approximately 6%.

The primary analysis will evaluate the deprescribing of any of the three potentially inappropriate medication classes targeted in the study. As a secondary analysis, we will stratify by targeted drug class.

#### Secondary outcomes

Dose reduction: Mean daily dose will be estimated using dates of dispensing, days of supply of dispensing and strength of the prescription. The proportion of patients with a dose reduction will be compared for the three arms using a binomial logistic regression model, before and after covariate adjustment. Secondary analyses will compare the three arms regarding continuous within-patient percentage change in dose using Kruskal-Wallis analysis of variance [24]. Measurement of dose reduction over 6-month follow-up requires participants to complete study follow-up, and censored participants will not contribute data to this analysis. However, we will adjust for correlates of missing data to reduce possible bias [25]. In secondary analyses including the full sample, we will consider joint modeling of monthly dose and time to censoring [26].

In analyses for polypharmacy prevalence, we will identify participants with evidence of dispensings of ≥5 unique medications over the respective 6-month study observation period. The three arms will be compared using binomial logistic regression. Alzheimer’s disease medications and the three potentially inappropriate medication classes will contribute to the measure of polypharmacy. The primary analysis for polypharmacy will include medications administered by any route and include topical or ocular medications. Additional analysis will assess polypharmacy based on oral medications only. A combination drug will be considered a single medication for the purpose of this analysis.

Additional analyses will examine the within-patient change in the number of inappropriate medications. As the observable change varies by pre-intervention number of inappropriate medications, we will accommodate this between-patient heterogeneity as follows: within-patient changes will be ranked separately by pre-intervention number of inappropriate medications, ranks will be transformed using normal scores to obtain comparable distributions across these strata, and treatment arms will be compared regarding transformed ranks [27] using analysis of covariance.

In analyses of other secondary outcomes, count-based outcomes such as per-patient number of emergency department visits will be analyzed using Poisson or negative binomial regression, accounting for zeros through piecewise zero-inflation modeling if warranted based on observed distributions [28]. Analyses will include a participant-specific offset equaling number of days observed prior to censoring (180 days for participants with no censoring), as well as adjustment for correlates of missing data. Mortality will be analyzed using survival analyses, including Kaplan-Meier curves, log-rank testing, and Cox proportional hazards modeling, accounting for censoring due to disenrollment. Among study participants who discontinue the targeted medication, we will determine if another agent within the targeted class was dispensed over the study observation period. Analyses will be analogous to those for dose reduction.

### Sample size

Our projected sample size for the trial is 14,442 patients, 4,814 patients in each of the three study arms. This represents the number of participants who were randomized. This is higher than our initial estimated minimal required sample size of 11,250, which was calculated assuming a power of 80%, overall Type I error rate of .05 with a Bonferroni correction for 3 pairwise comparisons of study arms (.05/3 = .0167), and 2-sided hypothesis testing.

Based on our prior analyses [29, 30], we anticipate death or health plan disenrollment in 9.9% of sampled patients within 3 months of the intervention (receipt of the letter), with the remaining 90.1% contributing data in the 6-month observation interval of interest (days 91–270 post-intervention)–that is, we anticipate a per-arm sample size of 4814 × 0.901 = 4337. For analyses of the primary outcome, absence of dispensing of targeted inappropriate prescription classes in days 91–270, we anticipate censoring in this interval for 13.5% of participants based on prior data. To make maximal use of observed data, we will use survival analysis to model time until an inappropriate prescription (a “failure”) in days 91–270. Detectable pairwise between-arm differences (e.g., between usual care and an intervention arm) are presented in Table 3 below for a range of possible percentages for “failure”, which means having a dispensing *for a targeted inappropriate medication*. For the range of “failure” percentages examined here, which reflect those seen in Martin et al, [17] detectable HRs range from 0.89 to 0.93. For example, if 75% of participants randomized to Arm 1 are observed to have a “failure” (prescription for a targeted inappropriate medication) by day 270, the detectable HR for an inappropriate prescription for Arm 2 versus Arm 1 is 0.9165, a 8.35% reduction in risk; the corresponding detectable “failure” probability for Arm 2 = 0.7193, a difference smaller–i.e., more precise–than that seen in Martin et al. [17]

**Table 3:** Detectable pairwise between-arm differences in hazard of inappropriate prescription classes in the 6-month study observation period.

Percent with inappropriate prescribing of targeted drug (“failure”), Study Arm 1	Detectable hazard ratio for inappropriate prescribing, Arm 2 versus Arm 1
40	.886
50	.898
60	.907
70	.914
75	.917
80	.919
85	.922
90	.924
95	.926
99	.927

For the secondary outcome of ≥50% reduction in dose, an estimated 3,752 participants per arm will contribute data. Detectable between-arm pairwise differences in the percentage with a dose reduction are presented in Table 4. For additional secondary outcomes, such as per-patient number of hospitalizations or ED visits, based upon prior data (mean of 0.35 hospitalizations per 6-month period and 0.4 ED visits per 6-month period), we will be able to detect rate ratios of 0.88 and 0.89, respectively (corresponding to intervention-related reductions of 11.4% and 10.7%), accounting for censoring due to death or disenrollment. For between-arm differences in mortality, assuming usual care 6-month mortality of 6.3%–likely an underestimate given a lag in ascertainment–and 7.6% censoring due to disenrollment based on information provided by the participating health plans, the detectable hazard ratio is 0.73 corresponding to per-arm survival percentages of 93.7% versus 95.3% (absolute difference of 1.63%).

**Table 4:** Detectable between-arm pairwise difference in percentage with ≥50% dose reduction.

% dose reduction, Arm 1	Detectable between-arm difference in % with dose reduction
1	0.62
2	0.92
3	1.15
4	1.34
5	1.51
10	2.13
15	2.57
20	2.90
25	3.16

### Ethical considerations

The Institutional Review Board (IRB) of the University of Massachusetts Chan Medical School (UMass Chan) and an independent Data Safety and Monitoring Board approved this clinical trial protocol (IRB#H00023453) on March 31, 2022. The IRB also approved a waiver of informed consent. The IRB approved Protocol is shown in S1 Protocol. The SPIRIT (Standard Protocol Items: Recommendations for Interventional Trials) Checklist is shown in S1 Checklist.

## Discussion

The findings of this large pragmatic trial of ~14,500 patients will compare the effect of a health plan based educational mailing to patients and their physicians, physicians alone, versus an arm that does not receive the intervention (usual care) on the cessation of potentially inappropriate prescribing of antipsychotics, strong anticholinergics, or sedative hypnotics among individuals with AD/ADRD. The primary outcome is the cessation of inappropriate prescribing of these medication classes at 6 months post-intervention, after a blackout period of 90 days. Secondary outcomes include dose-reduction of more than 50%, polypharmacy, several healthcare utilization measures, therapeutic switch, and mortality.

The findings of this health plan based large pragmatic trial will add to the evidence base on deprescribing generated from several large national and international trials but will place a specific focus on the AD/ADRD population [10, 11]. These findings have the potential to demonstrate the influence of stimulating conversations about appropriate medication use between caregivers of patients with dementia and their physicians.

As in any RCT, there are specific challenges and limitations in conducting pragmatic trials in a distributed research network of administrative claims database [15, 31]. Some challenges are common to any trial using administrative claims data. The assessment of medications using outpatient dispensing data does not account for over-the-counter medication use or medications paid for out of pocket that may result in potential misclassification of the outcome of polypharmacy. Although we plan to assess overall death as an outcome, the assessment of mortality is limited to in-hospital deaths as there is a long-time delay for complete information on out-of-hospital deaths in administrative claims data. There is also incomplete data on race and ethnicity which may impact our ability to characterize the cohort. Since the trial only include commercially insured participants, findings may not be generalizable to those that are uninsured.

## Conclusion

We anticipate this large pragmatic RCT will contribute to the evidence base on promoting deprescribing of potentially inappropriate medications among older adults with AD/ADRD. If successful, such light touch, inexpensive and highly scalable interventions have the potential to reduce the burden of potentially inappropriate prescribing for patients with dementia.

## Supporting information

S1 Table Domains of trial according to PRECIS framework. (DOCX)

S2 Table Targeted prescription drugs. (DOCX)

S1 Appendix Sample patient cover letter. (PDF)

S2 Appendix Fact sheets. (PDF)

S3 Appendix Pocket card. (PDF)

S4 Appendix Sample provider cover letter. (PDF)

S5 Appendix Deprescribing algorithms. (PDF)

S6 Appendix Tapering guides. (PDF)

S1 Protocol IRB approved protocol. (PDF)

S1 Checklist SPIRIT checklist. (PDF)
