# Supplementary material for: Developing a PRogram to Educate and Sensitize Caregivers to Reduce the Inappropriate Prescription Burden in the Elderly with Alzheimer’s Disease (D-PRESCRIBE-AD): Trial protocol and rationale of an open-label pragmatic, prospective randomized controlled trial
Source: PLoS One. 2024 Feb 12;19(2):e0297562. doi: 10.1371/journal.pone.0297562 (PMC10861034; doi:10.1371/journal.pone.0297562)
Supplement: S1 Table — (DOCX) [file pone.0297562.s001.docx]

**S1 Table. Domains of Trial According to PRECIS Framework**

|  | **PRECIS Criteria for Pragmatic Trials** | **D-PRESCRIBE-AD** |
| --- | --- | --- |
| Participants | All eligible participants enrolled, regardless of risk, responsiveness, comorbidities, or past compliance. | Adult health plan patients identified as AD/ADRD patients based on a diagnosis code or use of a medication for AD/ADRD will be automatically enrolled. |
| Intervention  Condition | Interventions are flexible, offering leeway in formulation and application. | Interventions allow flexibility according to patient and caregiver preferences. Varying levels of participation are expected. |
| Intervention  Practitioners | Interventions are applied to practitioners regardless of expertise of practice setting. | Intervention healthcare providers will not be specially selected based on experience with AD/ADRD patients or clinical setting. |
| Comparison  Condition | “Usual Practice” (or the best alternative) | Intervention will be compared to usual care. |
| Comparison  Practitioners | The control intervention is applied by the full range of clinicians in the full range of settings, with only ordinary attention to training, experience, and performance. | Usual care will be provided by real-world providers under usual practice conditions – with no additional training or supervision. |
| Follow-Up  Assessments | There are no research assessments; administrative databases are searched for outcomes. | All outcome data are collected from routinely collected health plan data. |
| Outcome  Definition | The primary outcome is objectively measured, meaningful to study participants, and does not depend on central adjudication. | Primary and secondary outcomes are defined by specific claims relating to medications and healthcare utilization – no clinical assessment is required. |
| Intervention  Compliance | There are no special strategies to improve compliance. | There will be no central clinical supervision of intervention providers. |
| Practitioner  Adherence | There are no special strategies to maintain practitioner adherence, and adherence is unobtrusively measured. | There will be no central clinical supervision of healthcare providers. |
| Primary  Comparison | The analysis includes all patients regardless of compliance. | All outcomes will be analyzed according to initial assignment  regardless of intervention participation or compliance. |
| **Additional Features of D-PRESCRIBE-AD** | | |
| Treatment  Assignment | Controls, prospectively identified, preferably by  randomization. | Eligible patients will be randomly assigned to one of the three intervention arms |
| Generalizability | The intervention should be tested across distinct health care settings. | The intervention will be evaluated in two large national health plans. |
| Leveraging an existing data infrastructure | D-PRESCRIBE-AD will be a large, pragmatic trial to test a health plan-based intervention taking advantage of the FDA Sentinel Initiative infrastructure. | Health plan data will be used for identification of participants and outcome assessment. |
