# Supplementary material for: Developing a PRogram to Educate and Sensitize Caregivers to Reduce the Inappropriate Prescription Burden in the Elderly with Alzheimer’s Disease (D-PRESCRIBE-AD): Trial protocol and rationale of an open-label pragmatic, prospective randomized controlled trial
Source: PLoS One. 2024 Feb 12;19(2):e0297562. doi: 10.1371/journal.pone.0297562 (PMC10861034; doi:10.1371/journal.pone.0297562)
Supplement: S2 Table — (DOCX) [file pone.0297562.s002.docx]

**S2 Table. Targeted Prescription Drugs**

| **Sedative/Hypnotics** |
| --- |
| Alprazolam |
| Clonazepam |
| Diazepam |
| Eszopiclone |
| Lorazepam |
| Temazepam |
| Zolpidem Tartrate |
| **Antipsychotics** |
| Aripiprazole |
| Haloperidol |
| Olanzapine |
| Quetiapine Fumarate |
| Risperidone |
| Ziprasidone HCl |
| **Strong Anticholinergics** |
| Amitriptyline HCl |
| Benztropine Mesylate |
| Cyclobenzaprine HCl |
| Cyproheptadine HCl |
| Dicyclomine HCl |
| Doxepin HCl |
| Fesoterodine Fumarate |
| Hydroxyzine HCl |
| Hydroxyzine Pamoate |
| Meclizine HCl |
| Nortriptyline HCl |
| Oxybutynin Chloride |
| Paroxetine HCl |
| Solifenacin Succinate |
| Tolterodine Tartrate |
