# Supplementary material for: Developing a PRogram to Educate and Sensitize Caregivers to Reduce the Inappropriate Prescription Burden in the Elderly with Alzheimer’s Disease (D-PRESCRIBE-AD): Trial protocol and rationale of an open-label pragmatic, prospective randomized controlled trial
Source: PLoS One. 2024 Feb 12;19(2):e0297562. doi: 10.1371/journal.pone.0297562 (PMC10861034; doi:10.1371/journal.pone.0297562)
Supplement: S1 Appendix — (PDF) [file pone.0297562.s003.pdf]

Health Plan Logo

[Date]

John Smith, managing your medications is important to your health

Reference to specific drug prescribed to patient.

It's necessary to talk with your doctor regularly about your medications to ensure they are still right for you. To help you do that, we've included information about **Lorazepam** which you are currently taking, so you can discuss this medication at your next doctor's visit.

Alert that drug may be inappropriate.

Lorazepam is sometimes prescribed to treat anxiety or sleep problems. Using this medication for a short time may be appropriate. However, taking it for a long time may lead to harmful side effects such as falls and fractures, dizziness, memory problems, or daytime fatigue. These side effects may be more common as you age or if you're taking the medication with other drugs. **Ask your doctor if Lorazepam is still the best treatment for you.**

Warning to NOT stop drug prematurely without talking to provider

***Important:* Do not stop or change this medication without talking to your doctor.**

[Health Plan Name] have partnered to bring you this information. If you do not wish to receive any more materials like this, please contact us at [plan contact information]

— Your <Health Plan> service team

Recommendation to discuss with provider and share with caregiver.

Share this information

Show this letter and the enclosed information sheet to your doctor at your next visit.

If you have someone who helps you with your medications, share these materials with them, too.

For more information

Phone:
xxx-xxx-xxxx (toll free)

Email:
email@healthplan.com

Website:
knowmymeds.org
