# Supplementary material for: Developing a PRogram to Educate and Sensitize Caregivers to Reduce the Inappropriate Prescription Burden in the Elderly with Alzheimer’s Disease (D-PRESCRIBE-AD): Trial protocol and rationale of an open-label pragmatic, prospective randomized controlled trial
Source: PLoS One. 2024 Feb 12;19(2):e0297562. doi: 10.1371/journal.pone.0297562 (PMC10861034; doi:10.1371/journal.pone.0297562)
Supplement: S2 Appendix — (PDF) [file pone.0297562.s004.pdf]

Are your medications still right for you?

As life changes, your medication needs may change as well. Medications that were once good for you may not be the best choice for you today.

The **medications** in this box are sometimes used **for anxiety or sleep:**

- Alprazolam (Xanax®)
- Clonazepam (Klonopin®)
- Diazepam (Valium®)
- Eszopiclone (Lunesta®)
- Lorazepam (Ativan®)
- Temazepam (Restoril®)
- Zolpidem (Ambien®)

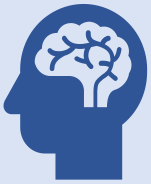

These medications can cause **side effects** including**:**

- falls and fractures
- dizziness
- worsened memory problems
- daytime fatigue
- dependence

As people age, they are more likely to experience side effects. Because of this, experts recommend that people on these medications talk to their healthcare provider about whether they should continue, reduce, or stop these medications.

Do not stop this medication before talking with your doctor. These medications must be reduced slowly. They should not be stopped suddenly. Stopping too quickly may cause problems.

Your doctor may suggest alternative medications or lifestyle changes that may help you.

What should you do?

- If someone helps you with your medications at home, share this information with them.
- **Bring this information sheet to your doctor.**
- Ask your doctor whether reducing or stopping this medication is the right choice for you.

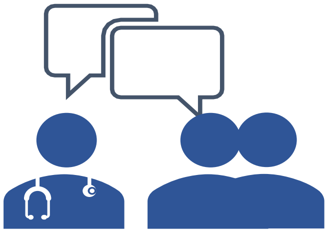

UMass Chan MEDICAL SCHOOL

This information sheet and other resources can be found at **KnowMyMeds.org.**
It was produced by clinicians and researchers at the UMass Chan Medical School, adapted from materials developed by B. Farrell of the Bruyère Research Institute and C. Tannenbaum of the Université de Montréal. This work was funded by the National Institute on Aging.

Are your medications still right for you?

As life changes, your medication needs may change as well. Medications that were once good for you may not be the best choice for you today.

The medications in this box are called **antipsychotic medications.**

- Aripiprazole (Abilify®)
- Haloperidol (Haldol®)
- Olanzapine (Zyprexa®)
- Quetiapine (Seroquel®)
- Risperidone (Risperdal®)
- Ziprasidone (Geodon®)

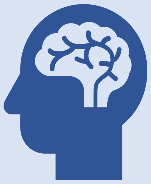

Antipsychotic medications can cause **side effects including:**

- balance problems
- falls
- spasms
- tremors
- jerky movements
- fatigue
- dry mouth

As people age, they are more likely to experience side effects. Because of this, experts recommend that people on these medications talk to their doctor about whether they should continue, reduce, or stop these medications.

Do not stop this medication before talking with your doctor. These medications must be reduced slowly. They should not be stopped suddenly. Stopping too quickly may cause problems.

Your doctor may suggest alternative medications or lifestyle changes that may help you.

What should you do?

- If someone helps you with your medications at home, share this information with them.
- **Bring this information sheet to your doctor.**
- Ask your doctor whether reducing or stopping this medication is the right choice for you.

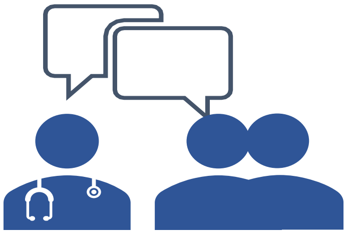

UMass Chan MEDICAL SCHOOL

This information sheet and other resources can be found at **KnowMyMeds.org.**
It was produced by clinicians and researchers at the UMass Chan Medical School, adapted from materials developed by B. Farrell of the Bruyère Research Institute and C. Tannenbaum of the Université de Montréal. This work was funded by the National Institute on Aging.

Are your medications still right for you?

As life changes, your medication needs may change as well. Medications that were once good for you may not be the best choice for you today.

These medications can cause **anticholinergic side effects.**

- Amitriptyline (Elavil®)
- Benztropine (Congentin®)
- Cyclobenzaprine (Flexeril®)
- Cyproheptadine (Periactin®)
- Dicyclomine (Bentyl®)
- Doxepin (Sinequan®)
- Fesoterodine (Toviaz®)
- Hydroxyzine (Atarax®)
- Hydroxyzine Pamoate (Vistaril®)
- Meclizine (Antivert®)
- Nortriptyline (Aventyl®/Pamelor®)
- Oxybutynin (Ditropan®)
- Paroxetine (Paxil®)
- Solifenacin (VESIcare®)
- Tolterodine (Detrol®)
- Trospium (Sanctura®)

Anticholinergic side effects include:

- dry mouth
- blurred vision
- drowsiness
- constipation
- urinary problems

As people age, they are more likely to experience side effects. Because of this, experts recommend that people on these medications talk to their doctor about whether they should continue, reduce, or stop these medications.

Do not stop this medication before talking with your doctor. Certain medications must be reduced slowly and should not be stopped suddenly. Stopping too quickly may cause problems.

Your doctor can suggest alternative medications or lifestyle changes that may help you

What should you do?

- If someone helps you with your medications at home, share this information with them.
- **Bring this information sheet to your doctor.**
- Ask your doctor whether reducing or stopping this medication is the right choice for you.

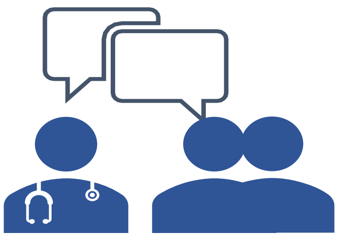

UMass Chan MEDICAL SCHOOL

This information sheet and other resources can be found at **KnowMyMeds.org.**
It was produced by clinicians and researchers at the UMass Chan Medical School, adapted from materials developed by B. Farrell of the Bruyère Research Institute and C. Tannenbaum of the Université de Montréal. This work was funded by the National Institute on Aging.
