# Supplementary material for: Developing a PRogram to Educate and Sensitize Caregivers to Reduce the Inappropriate Prescription Burden in the Elderly with Alzheimer’s Disease (D-PRESCRIBE-AD): Trial protocol and rationale of an open-label pragmatic, prospective randomized controlled trial
Source: PLoS One. 2024 Feb 12;19(2):e0297562. doi: 10.1371/journal.pone.0297562 (PMC10861034; doi:10.1371/journal.pone.0297562)
Supplement: S3 Appendix — (PDF) [file pone.0297562.s005.pdf]

Questions to ask your doctor

- Is this medication still right for me?
- Are there lifestyle changes that I could try instead?
- If I don't need this medication, can we make a plan to discontinue it?

know mymeds

Always consult with your doctor before making any changes to your medications.
