# Supplementary material for: Developing a PRogram to Educate and Sensitize Caregivers to Reduce the Inappropriate Prescription Burden in the Elderly with Alzheimer’s Disease (D-PRESCRIBE-AD): Trial protocol and rationale of an open-label pragmatic, prospective randomized controlled trial
Source: PLoS One. 2024 Feb 12;19(2):e0297562. doi: 10.1371/journal.pone.0297562 (PMC10861034; doi:10.1371/journal.pone.0297562)
Supplement: S5 Appendix — (PDF) [file pone.0297562.s007.pdf]

## Patient taking an anticholinergic medication for urinary incontinence

- Adverse effects: dry mouth, blurred vision, constipation
- Symptoms have not improved
- Other underlying causes of incontinence (diuretic use, diabetes, urinary tract infection)

### Engage patients/caregivers

- Discuss potential risks, benefits, and withdrawal plan

### Recommend deprescribing

### Taper slowly over 3-4 weeks

### Monitor weekly for worsening of symptoms

**Use non-drug approaches to manage incontinence:**

- Symptom diary
- Reduce fluid intake
- Bladder training
- Timed voiding
- Pelvic floor exercises

**If symptoms relapse:**

- Consider reinitiating therapy

---

- Symptoms have improved and no significant adverse effects

### Continue

UMass Chan MEDICAL SCHOOL

This information sheet was produced by clinicians and researchers at the UMass Chan Medical School, funded by the National Institute on Aging.

know my meds

# Antipsychotic Deprescribing Algorithm

## Why is patient taking an antipsychotic?

- Psychosis, aggression, agitation (behavioral and psychological symptoms of dementia) treated ≥ 3 months (symptoms controlled, or no response to therapy)

- Primary insomnia treated for any duration or secondary insomnia where underlying comorbidities are managed

- Schizophrenia
- Schizo-affective disorder
- Bipolar disorder
- Acute delirium
- Tourette's syndrome
- Tic disorders
- Autism
- Less than 3 months duration of psychosis in dementia
- Intellectual disability
- Developmental delay
- Obsessive-compulsive disorder
- Alcoholism
- Parkinson's disease psychosis
- Adjunct treatment of Major Depressive Disorder

## Recommend deprescribing

### Taper and stop antipsychotic

Slowly in collaboration with patient and/or caregiver; e.g., 25%-50% dose reduction every 1-2 weeks

### Stop antipsychotic

Good practice recommendation

### Continue antipsychotic

Or consult psychiatrist if considering deprescribing

### Monitor every 1-2 weeks for duration of tapering

**Expected benefits:**

- May improve alertness, gait, reduce falls, or extrapyramidal symptoms

**Adverse drug withdrawal events** (closer monitoring for those with more severe baseline symptoms):

- Psychosis, aggression, agitation, delusions, hallucinations

**If behavioral and psychological symptoms of dementia relapse:**

**Consider:**

- Non-drug approaches (e.g. music therapy, behavioral management strategies)

**Restart an antipsychotic drug:**

- Restart antipsychotic at lowest dose possible if resurgence of behavioral and psychological symptoms of dementia with re-trial of deprescribing in 3 months
- At least 2 attempts to stop should be made

**If insomnia relapses, recommend non-drug approaches to patients:**

1. Go to bed only when sleepy
2. Do not use bed or bedroom for anything but sleep (or intimacy)
3. If not asleep within about 20-30 min at the beginning of the night or after an awakening, exit the bedroom
4. If not asleep within 20-30 min on returning to bed, repeat #3
5. Use alarm to awaken at the same time every morning
6. Do not nap
7. Avoid caffeine after noon
8. Avoid exercise, nicotine, alcohol, and big meals within 2 hrs of bedtime

UMass Chan MEDICAL SCHOOL

This information sheet and other resources can be found at **KnowMyMeds.org.** It was produced by clinicians and researchers at the UMass Chan Medical School, adapted from materials developed by B. Farrell of the Bruyère Research Institute and C. Tannenbaum of the Université de Montréal. This work was funded by the National Institute on Aging.

know mymeds

# Sedative/Hypnotic Deprescribing Algorithm

## Why is patient taking a sedative/hypnotic?

If unsure, find out if history of anxiety, past psychiatric consult, whether may have been started in hospital for sleep, or for grief reaction.

- Insomnia on its own OR insomnia where underlying comorbidities managed
  For those ≥ 65 years of age: taking sedative/hypnotic regardless of duration (avoid as first therapy in older people)
  For those 18-64 years of age: taking sedative/hypnotic > 4 weeks

## Engage patients

- Discuss potential risks, benefits, withdrawal plan, symptoms and duration

## Recommend deprescribing

## Taper and then stop sedative/hypnotic

- Taper slowly in collaboration with patient, for example ~25% every two weeks and, if possible, 12.5% reductions near end and/or planned drug-free days

## Monitor every 1-2 weeks for duration of tapering

Expected benefits:

- May improve alertness, cognition, daytime sedation and reduce falls

Withdrawal symptoms:

- Insomnia, anxiety, irritability, sweating, gastrointestinal symptoms (all usually mild and last for days to a few weeks)

---

- Other sleeping disorders (e.g., restless legs)
- Unmanaged anxiety, depression, physical or mental condition that may be causing or aggravating insomnia
- Alcohol withdrawal

## Continue sedative/hypnotic

- Minimize use of drugs that worsen insomnia (e.g., caffeine, alcohol etc.)
- Treat underlying condition
- Consider consulting psychologist or psychiatrist or sleep specialist
- Use lowest possible effective dose

---

- Use non-drug approaches to manage insomnia

If symptoms relapse:

Consider

- Maintaining current sedative/hypnotic dose for 1-2 weeks, then continue taper at slow rate

Alternate drugs

- Other medications have been used to manage insomnia. Assessment of their safety and effectiveness is beyond the scope of this algorithm.

# Sedative/Hypnotic Deprescribing Advice

## Engaging patients and caregivers

**Patients should understand:**

- The rationale for deprescribing (associated risks of continued sedative/hypnotic use, reduced long-term efficacy)
- Withdrawal symptoms (insomnia, anxiety) may occur but are usually mild, transient and short-term (days to a few weeks)
- They are part of the tapering plan, and can control tapering rate and duration

## Tapering doses

- No published evidence exists to suggest switching to long-acting sedatives/hypnotics reduces incidence of withdrawal symptoms or is more effective than tapering shorter-acting sedatives/hypnotics
- If dosage forms do not allow 25% reduction, consider 50% reduction initially using drug-free days during latter part of tapering, or switch to lorazepam or oxazepam for final taper steps

## Behavioral Management

1. Go to bed only when sleepy
2. Do not use bed or bedroom for anything but sleep (or intimacy)
3. If not asleep within about 20-30 min at the beginning of the night or after an awakening, exit the bedroom
4. If not asleep within 20-30 min on returning to bed, repeat #3
5. Use alarm to awaken at the same time every morning
6. Do not nap
7. Avoid caffeine after noon
8. Avoid exercise, nicotine, alcohol, and big meals within 2 hrs of bedtime

UMass Chan MEDICAL SCHOOL

This information sheet and other resources can be found at **KnowMyMeds.org.**
It was produced by clinicians and researchers at the UMass Chan Medical School, adapted from materials developed by B. Farrell of the Bruyère Research Institute and C. Tannenbaum of the Université de Montréal. This work was funded by the National Institute on Aging.

know mymeds
