# Supplementary material for: Developing a PRogram to Educate and Sensitize Caregivers to Reduce the Inappropriate Prescription Burden in the Elderly with Alzheimer’s Disease (D-PRESCRIBE-AD): Trial protocol and rationale of an open-label pragmatic, prospective randomized controlled trial
Source: PLoS One. 2024 Feb 12;19(2):e0297562. doi: 10.1371/journal.pone.0297562 (PMC10861034; doi:10.1371/journal.pone.0297562)
Supplement: S6 Appendix — (PDF) [file pone.0297562.s008.pdf]

Medication Tapering Plan

Talk to your doctor, nurse or pharmacist before making any changes to your medication.

Patient Name: ____________ Doctor: __________ Medication: _____________

Tapering Schedule							
**WEEK OF:**	**SU**	**MO**	**TU**	**WE**	**TH**	**FR**	**SA**
1. __/__	◒ ☐	● ☐	◒ ☐	● ☐	◒ ☐	● ☐	◒ ☐
2. __/__	◒ ☐	● ☐	◒ ☐	● ☐	◒ ☐	● ☐	◒ ☐
3. __/__	◒ ☐	◒ ☐	◒ ☐	◒ ☐	◒ ☐	◒ ☐	◒ ☐
4. __/__	◒ ☐	◒ ☐	◒ ☐	◒ ☐	◒ ☐	◒ ☐	◒ ☐
5. __/__	◒ ☐	✕ ☐	◒ ☐	✕ ☐	◒ ☐	✕ ☐	◒ ☐
6. __/__	◒ ☐	✕ ☐	◒ ☐	✕ ☐	◒ ☐	✕ ☐	◒ ☐
7. __/__	✕ ☐	✕ ☐	✕ ☐	✕ ☐	✕ ☐	✕ ☐	✕ ☐
8. __/__	✕ ☐	✕ ☐	✕ ☐	✕ ☐	✕ ☐	✕ ☐	✕ ☐

What the symbols mean:

● Full dose ___________

◒ Half dose ___________

◔ Quarter of a dose ___________

✕ No dose

Talk to your doctor, nurse or pharmacist before changing or stopping your medication.

This guide for antipsychotic medication and other resources can be found at **KnowMyMeds.org.**
It was produced by clinicians and researchers at the UMass Chan Medical School, adapted from materials developed by B. Farrell of the Bruyère Research Institute and C. Tannenbaum of the Université de Montréal. This work was funded by the National Institute on Aging.

Medication Tapering Plan

Talk to your doctor, nurse or pharmacist before making any changes to your medication.

Patient Name: ______________ Doctor: ____________ Medication: ________________

Tapering Schedule							
**WEEK OF:**	**SU**	**MO**	**TU**	**WE**	**TH**	**FR**	**SA**
**1.** __/__	☐	☐	☐	☐	☐	☐	☐
**2.** __/__	☐	☐	☐	☐	☐	☐	☐
**3.** __/__	☐	☐	☐	☐	☐	☐	☐
**4.** __/__	☐	☐	☐	☐	☐	☐	☐
**5.** __/__	☐	☐	☐	☐	☐	☐	☐
**6.** __/__	☐	☐	☐	☐	☐	☐	☐
**7.** __/__	☐	☐	☐	☐	☐	☐	☐
**8.** __/__	☐	☐	☐	☐	☐	☐	☐
**9.** __/__	☐	☐	☐	☐	☐	☐	☐
**10.** __/__	☐	☐	☐	☐	☐	☐	☐
**11.** __/__	☐	☐	☐	☐	☐	☐	☐
**12.** __/__	☐	☐	☐	☐	☐	☐	☐
**13.** __/__	☐	☐	☐	☐	☐	☐	☐

14. __/__	◔ ☐	◔ ☐	◔ ☐	◔ ☐	◔ ☐	◔ ☐	◔ ☐
15. __/__	✕ ☐	◔ ☐	✕ ☐	✕ ☐	◔ ☐	✕ ☐	◔ ☐
16. __/__	✕ ☐	◔ ☐	✕ ☐	✕ ☐	◔ ☐	✕ ☐	◔ ☐
17. __/__	✕ ☐	✕ ☐	✕ ☐	✕ ☐	✕ ☐	✕ ☐	✕ ☐
18. __/__	✕ ☐	✕ ☐	✕ ☐	✕ ☐	✕ ☐	✕ ☐	✕ ☐

What the symbols mean:

● Full dose ____________

◓ Half dose ____________

◔ Quarter dose ____________

✕ No dose

Talk to your doctor, nurse or pharmacist before changing or stopping your medication.

This guide for sedative-hypnotic medication and other resources can be found at **KnowMyMeds.org.**
It was produced by clinicians and researchers at the UMass Chan Medical School, adapted from materials developed by B. Farrell of the Bruyère Research Institute and C. Tannenbaum of the Université de Montréal. This work was funded by the National Institute on Aging.
