# Supplementary material for: Developing a PRogram to Educate and Sensitize Caregivers to Reduce the Inappropriate Prescription Burden in the Elderly with Alzheimer’s Disease (D-PRESCRIBE-AD): Trial protocol and rationale of an open-label pragmatic, prospective randomized controlled trial
Source: PLoS One. 2024 Feb 12;19(2):e0297562. doi: 10.1371/journal.pone.0297562 (PMC10861034; doi:10.1371/journal.pone.0297562)
Supplement: S1 Protocol — (PDF) [file pone.0297562.s009.pdf]

INVESTIGATOR STUDY PLAN - REQUIRED

1. TITLE

D- PRESCRIBE-AD (The Developing a PRogram to Educate and Sensitize Caregivers to Reduce the Inappropriate Prescription Burden in Elderly with Alzheimer's Disease Study)

2. EXTERNAL IRB REVIEW HISTORY*

N/A

3. PRIOR APPROVALS:

Health plans will execute a reliance agreement prior to any work and abide by the approved ISP.

4. OBJECTIVES*

Enhancing patient/caregiver communication with the healthcare provider about medications may help reduce inappropriate prescribing to persons with AD/ADRD. The overarching goal of our proposal is to develop, implement, and evaluate the effect of a patient/caregiver-centered, multifaceted educational intervention on inappropriate prescribing in patients with AD/ADRD. The Developing a PRogram to Educate and Sensitize Caregivers to Reduce the Inappropriate Prescription Burden in Elderly with Alzheimer's Disease Study (D-PRESCRIBE-AD) will be a large, randomized, pragmatic trial to test a health plan-based intervention leveraging the NIH Collaboratory's Distributed Research Network, which uses the Food and Drug Administration (FDA) Sentinel Initiative infrastructure. In this study, we will enroll community dwelling AD/ADRD patients (based on a diagnosis of AD/ADRD or use of a medication for AD), who have evidence of inappropriate prescribing. We will evaluate the effect of educational interventions designed to stimulate patient/caregiver-provider communication about medication safety (versus usual care) on the cessation of inappropriate prescribing, **the primary outcome** of this study. The educational intervention will be an adaptation of an intervention proven effective in reducing the use of inappropriate medications in older adults, modified for the AD/ADRD population and their caregivers.

Our study will be conducted in two national health plans and will represent a substantial scaling-up of prior educational interventions focused on inappropriate prescribing. The study design will be a **prospective, randomized, "open-label" educational intervention trial with three arms: (1) a combined patient/caregiver and provider educational intervention; (2) a provider only educational intervention; and (3) usual care**.

It has two sequential phases. We have conducted a one- year R61 planning phase to precede a four-year R33 implementation phase. During the one-year R61 planning phase, we have finalized the intervention and conducted feasibility testing, and stakeholder engagement and met the required milestones. The aims of R33 Implementation Phase are as follows:

Aim 1: To assess the impact of the patient/caregiver educational intervention on the primary outcome of cessation of inappropriate prescribing among AD/ADRD patients, employing a prospective, randomized trial design with three arms: (1) a combined patient/caregiver and provider educational intervention; (2) a provider only educational intervention; and (3) usual care. Secondary outcomes will include any dose reduction of inappropriate medications,

prevalence of polypharmacy; rates of emergency room visits; rates of hospitalizations; rates of non-acute institutional stays (e.g., skilled nursing facilities); overall health care utilization (number of outpatient visits, days hospitalized, emergency department visits, and non-acute institutional days); inpatient mortality, and switching within classes.

Aim 2: To create: (1) a plan for disseminating study findings to stakeholders who might implement the intervention or make decisions about its future use; and (2) an implementation toolkit for health plans and health systems wishing to implement the intervention.

5. Background*

Polypharmacy, commonly defined as use of five or more medications, is directly associated with multimorbidity and is prevalent among persons with AD/ADRD. Polypharmacy substantially increases the likelihood of being exposed to inappropriate medications and the likelihood that inappropriate medications will lead to adverse drug events, falls, worsening cognitive impairment, and emergency hospitalizations. Inappropriate prescribing includes the use of medications that may no longer be necessary or that may increase the risk of harm. While the characterization of a medication as "inappropriate" might be considered by some as absolutist, for the purpose of this application, the designation "inappropriate prescribing" or "inappropriate medication" indicates the need to carefully assess the risks of continued use versus the benefits. In a sense, inappropriate prescribing can be thought of as a "morbidity multiplier," increasing overall symptom burden, and adversely affecting health-related quality of life and function. Certain drug categories, such as sedative/hypnotics, antipsychotic medications, and strong anticholinergic agents, pose special risks for older adults. Patients with AD/ADRD are at particularly increased risk for inappropriate prescribing due to high levels of multimorbidity and polypharmacy, superimposed on the challenges and complexities of their care. Patient/caregiver communication with the healthcare provider regarding medications is often suboptimal. Addressing this challenge requires an intervention in which patients, caregivers, providers, and health systems can play an active role.

Patients and family caregivers have important insights into their care, but often do not speak up about these concerns. Consequently, if healthcare providers are unaware of these concerns, they are unable to correct misperceptions or to address and correct actual care breakdowns, including medication safety issues. Some healthcare systems have sought to address this challenge, through a campaign called "*We Want to Know*" (conceived by Dr. Kathleen Mazor, a co-investigator on this application) that seeks to address patient concerns and questions about their care in real-time. While this initiative has been focused on engaging patients and families to speak up if they have a concern about their care in the hospital, *"We Want to Know"* serves as a model for activating patients and caregivers to engage providers with the purpose of identifying and addressing situations like inappropriate prescribing. The Alzheimer's Association has also sought to activate patients and their caregivers through the use of a "Doctor's Visit Checklist" that includes: (1) taking a list of concerns to the visit with the healthcare provider; (2) taking a medication list or medicine bottles to the visit; and (3) asking questions until you understand everything.

Several direct-to-patient educational efforts have been shown to be effective in improving the quality and safety of pharmacotherapy. Dr. Cara Tannenbaum, a consultant on our application, has led a number of Canadian studies focused on reducing inappropriate prescribing to older

adults through direct patient education designed to elicit shared decision-making. Most relevant to our proposed study, Dr. Tannenbaum's team conducted a consumer-focused educational intervention, targeting the inappropriate prescribing of several Beers Criteria medications in older adults (D-PRESCRIBE). In D-PRESCRIBE, educational materials were distributed by pharmacy-based pharmacists by mail or in-person, and contained information about why the medication may be inappropriate, potential alternative treatment options, and tapering protocols for sedative/hypnotics. In this modest-sized trial, at 6 months, 106 of 248 patients (43%) in the intervention group no longer filled prescriptions for inappropriate medication compared with 29 of 241 (12%) in the control group (risk difference 31% [95% confidence interval, 23% to 38%]). We will adapt Dr. Tannenbaum's proven approach, modified specifically for the AD/ADRD population and their caregivers, for implementation in two national health plans. Our efforts will represent a substantial scaling-up of prior efforts focused on reducing inappropriate prescribing.

Deprescribing is the clinically supervised process of stopping medications that could cause harm or that no longer provide benefits that outweigh potential risks. It is not an action that the patient and/or caregiver takes independent of the prescriber, as it occurs under the guidance and direction of the healthcare provider. Recognizing the multiplicity of factors that influence and challenge deprescribing efforts, Linsky and colleagues recently published a unifying deprescribing conceptual framework, generalizable across healthcare settings, to advance the science of deprescribing research and to foster the design, conduct, and dissemination of deprescribing trials.[18] Importantly, this new conceptual framework emphasizes the roles of the patient/caregiver, prescriber, and healthcare system, all of which influence the decision and ability to deprescribe. Linsky's framework emphasizes that the deprescribing process (including the decision to deprescribe) is ideally shared by patients and healthcare providers. It takes into account effects and measures, including process measures of the performance of the intervention, and outcomes including ongoing use of inappropriate medications, hospitalization, and mortality. Linsky and colleagues also recognize the challenges and delays involved in disseminating and implementing effective interventions, highlighting that the findings of deprescribing studies "will be limited in impact unless successful approaches are broadly taken up across healthcare systems." The figure below adapts and applies Linsky's deprescribing conceptual framework to our proposed D-PRESCRIBE-AD Study. **(Figure 1)**

Figure 1. Deprescribing Framework

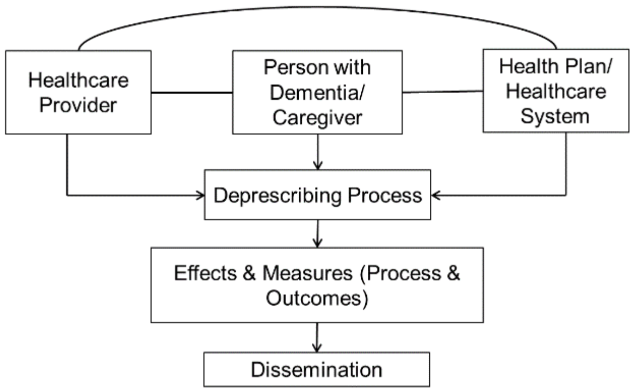

Our overarching **hypothesis is that inappropriate prescribing of antipsychotics, sedative-hypnotics and strong anticholinergics in AD/ADRD patients can be addressed through enhanced communication between the patient/caregiver and the provider, facilitated by the patient's health plan**. The evidence of medication-related morbidity as a public health issue justifies large scale efforts to reduce inappropriate prescribing in vulnerable patient populations, such as those with AD/ADRD. Evidence also exists that simple direct-to-patient educational interventions can impact positively on medication use patterns, including discontinuation of potentially harmful therapies. However, existing evidence certainly does not prove effectiveness, or even the feasibility, of large-scale, simple educational interventions targeting persons with AD/ADRD and caregivers, in addition to their healthcare providers. New research is needed to: (1) demonstrate the feasibility of population-based outreach to AD/ADRD patients at high-risk for inappropriate prescribing and their family caregivers; (2) demonstrate the feasibility and effectiveness of a low-intensity educational intervention focused on reducing inappropriate prescribing involving AD/ADRD patients, their family caregivers, and healthcare providers; (3) demonstrate the value and efficiency of capitalizing on routinely collected health plan data to identify high-risk populations and to assess primary and secondary outcomes; and (4) demonstrate the potential to adapt, spread, and scale-up a proven intervention (D-PRESCRIBE[3]) to address inappropriate prescribing at a national level.

Our proposed D-PRESCRIBE-AD study will take advantage of the **NIH Collaboratory Distributed Research Network, which uses the FDA Sentinel Initiative infrastructure**. The FDA Sentinel Initiative has previously initiated proof-of concept endeavors employing direct-to-patient strategies using the Sentinel infrastructure and the network of participating health plans.

The proposed research will represent a rigorous evaluation of a large scale, health plan-based, educational intervention to improve medication safety and reduce preventable medication-related morbidity among high-risk AD/ADRD patients. By design, the proposed intervention will be transportable to other large health plans and healthcare systems.

6. Inclusion and Exclusion Criteria*

We will identify trial-eligible patients with Alzheimer's disease or Alzheimer's disease related dementia (AD/ADRD) and recent evidence of targeted inappropriate prescribing of antipsychotics, sedative/hypnotics, and strong anticholinergics in the two participating health plans (Humana and HealthCore). The cohort will be restricted to research eligible, non-administrative services only members at the two participating health plans and those **NOT** on the do-not-call list.

Patient Inclusion Criteria:

1. Diagnosis of AD/ADRD based on a modified list of the Chronic Conditions Data Warehouse codes, or treatment with a pharmacologic therapy used for AD (e.g., donepezil, rivastigmine, galantamine, and memantine) in the 365 days prior to or on cohort entry date.
    - The two AD/ADRD ICD-10 diagnosis codes must be ≥7 day apart and at least one of the codes is within 365 days of the cohort entry date.

- Treatment is defined as exposure to ADRD drug based on either: (1) days supply of one or more dispensing, or (2) a dispensing in the 365 days prior to cohort entry date

2. Evidence of inappropriate prescribing with the selected inappropriate medication classes including antipsychotics, sedative-hypnotics, and strong anticholinergics within the past 6 months prior to and through the cohort entry date
3. Age ≥50 years of age as of cohort entry date
4. Continuous medical and pharmacy insurance coverage for at least the prior year

<u>Exclusion Criteria.</u>
An individual who meets any of the following criteria will be excluded from participation in this study:

1. Evidence of a recent institutional stay encounter in a skilled nursing facility, hospice, rehab center, nursing home, residential, overnight non-hospital dialysis and other non-hospital stays within the previous 90 days prior to or on cohort entry date.
2. Incomplete/missing prescriber ID or incomplete contact information for either patient or prescribing provider.
3. On "do not contact" list

<u>Provider Inclusion Criteria:</u>

1. Providing treatment to any patient enrolled in the study.

<u>Vulnerable Populations:</u>

1. Children will not be included in this study as the disease under study primarily afflicts the adult population. The inclusion criteria includes an age of 50 years or greater.
2. We will not knowingly collect data on prisoners. If the health plan becomes aware of a member's prisoner-status, we will ask them to eliminate those data from the queries.
3. It is possible that pregnant women will be included as this is not explicitly an exclusion criterion. However, this is unlikely as our age restriction is over 50.
4. Data related to adults unable to consent will likely be included in this population.
5. Non-English-speaking subjects will be included in this population.

See also section #18 Vulnerable Populations for more information on protections of included vulnerable populations.

<u>Advisors</u>

Stakeholder Panel. We will identify and engage a diverse Stakeholder Panel which will include approximately 3 patients and/or family caregivers, 3 health care providers who care for patients with ADRD, and 2 health plan leaders. **These individuals are not considered study subjects.** They will meet periodically and share their expertise on aspects of the study design and materials. The identification of the health plan leaders will be facilitated by the two participating health plan sites - Humana and Health Core.

Advisory Committee. The investigator team will convene an advisory committee, made up of several nationally recognized leaders with expertise in how older adults interact with the health system (including investigators and a family caregiver advocate), to advise the PI and the research team throughout the project. All Advisory Committee members will participate in quarterly meetings/conference calls, as well as reviewing materials and responding to requests for feedback as needed. **These individuals are not considered study subjects.**

7. STUDY-WIDE NUMBER OF SUBJECTS*

We will sequentially implement **two separate pragmatic trials** (Implementation Phase 1 and Implementation Phase 2), the first enrolling up to 15,000 patients, with the second trial to be adapted based on the findings and experience gained in the first trial. Adaptations could include dropping the provider only arm and/or or further limiting the classes of inappropriate medications targeted.

Note: We will submit a modification for approval when we know the number of subjects to be recruited for the second trial.

8. STUDY-WIDE RECRUITMENT METHODS*

The patients in D-PRESCRIBE-AD will be randomly selected from the membership of the two participating health plans (HealthCore/Anthem and Humana) who meet inclusion criteria determined through health plan administrative claims data. Each participating health plan will send approved intervention materials to their respective patients and providers as appropriate according to their random group assignment. Providers and patients/caregivers will receive applicable educational materials through a one-time mailing at trial start. All patients who are assigned to a group will be considered enrolled. Participants enrolled in the clinical trial will not receive a stipend.

Trial participants will only be contacted once as detailed above. Outcomes of interest will be provided by the health plan to the Analytic Coordinating Center, with identifiable patient-level data removed, relating to the 6-month observation period that follows a 3-month lag period after the initial mailing. Retention of participants will be determined by their continued enrollment with their respective health plan, and as such, the study team will not have a specific plan for retention.

9. STUDY TIMELINES*

Timeline	Year 1				Year 2				Year 3				Year 4				Year 5			
	Q1	Q2	Q3	Q4	Q1	Q2	Q3	Q4	Q1	Q2	Q3	Q4	Q1	Q2	Q3	Q4	Q1	Q2	Q3	Q4
**R61 Planning Phase**																				
**R33 Implementation Phase**																				
**Implementation Phase 1**																				
Identification of study cohort					X															
Randomization of subjects					X															
Mailing of Intervention Materials						X														
6-month observation period								X	X											
Analysis of 1st Mailing												X								
**Implementation Phase 2**																				
Identify Study Cohort #2												X								
Randomization #2												X								
Mailing of Intervention Materials													X							
6-month observation period															X	X				
Analysis of 2nd Mailing																			X	
Meet with DSMB		X		X		X		X		X		X		X		X		X		X
Prepare manuscripts	Ongoing																			
**Dissemination**															X	X	X	X	X	X
Engage Advisors/ Stakeholders	Throughout project																			

Data collection will take 24 months overall from the cohort identification mailing until statistical analysis of data for the first trial. The observation period will extend 9 months (following a 3-month "blackout" period following the mailing) for each participant.

10. STUDY ENDPOINTS*

Primary Endpoint: We will evaluate the effect of educational interventions designed to stimulate patient/caregiver-provider communication about medication safety (versus usual care) on the primary outcome. The primary outcome will be defined as absence of any dispensing of the targeted inappropriate prescription class from day 91 to day 270 during the 9 months following receipt of intervention.

Secondary Endpoints: Secondary outcomes are listed below.

These will also be assessed specific to the 6-month observation period (days 91-270 following mailing/intervention) based on health plan claims data including:

1. Any dose reduction (defined as > 50% reduction in dose of the selected inappropriate medication), assessed at the participant level using health claims data (outpatient dispensing).
2. Decrease in the proportion of patients with polypharmacy. (defined as >5 active prescriptions for different agents)
3. Decline in the rates of: emergency room visits; rates of hospitalizations; rates of non-acute institutional stays (e.g., skilled nursing facilities); and overall health care utilization (number of outpatient visits, days hospitalized, emergency department visits, and non- acute institutional days).
4. Decline in-hospital all-cause mortality. (We can only study in-hospital all-cause mortality due to a delay in receipt of comprehensive death data.)
5. Switching within classes

We will use administrative claims data to identify encounters of interest (ED visits, hospitalizations, non-acute institutional stays, outpatient visits) and only assess oral formulations for medications.

11. PROCEDURES INVOLVED*

A program, developed by the analytic coordinating center (HPHCI), will be used by the participating health plans to identify their patients who meet eligibility criteria for the trial.

As there may be the potential for within-provider "contamination," such that some providers would be treating patients who are randomized to different study arms, for any provider with more than one eligible patient, only one patient will be randomly enrolled. The list of eligible patients will stay with the health plan, and identifiable patient-level data will not be shared with the analytic coordinating center. The health plan will remove any individuals who cannot be included in research studies for any reason. The list of randomized patients will include the primary care provider based on the most recent data used for cohort identification. As with the patient list, the provider list will not be shared by the participating health plans, as each participating health plan will be mailing the intervention materials to their respective patients/caregivers and providers.

An electronic program, developed by the central coordinating center (Harvard Pilgrim), will be used by the participating health Plans to identify their eligible member-patients who meet the inclusion and exclusion criteria for this trial. A list of eligible member-patients will stay at each health plan, and identifiable patient level data will not be shared with the central coordinating center (Harvard Pilgrim) or the statistical analysis center (University of Massachusetts Chan Medical School) (UMass Chan). The health plans will remove any individuals who cannot be included in research studies, which includes certain Centers for Medicare and Medicaid Services and Administrative Services Only patients. There are several other reasons that could prevent mailing of the intervention, to members and/or their providers (list may not be exhaustive):

- Member is now deceased
- Member disenrolls from health plan
- Member transitions to a plan that does not allow for inclusion in research
- Member has an incorrect/missing/bad address
- Member is added to a do-not-contact list at the health plan

The list of randomized individuals will include the provider on each patient's most recent encounter with an AD/ADRD as of the last date in the current approved data used for cohort identification. When the identified provider is an individual, this provider will receive the provider intervention materials. As with the member-patient list, the provider list will be kept by each Health Plan and not shared, as each Health Plan will be mailing the intervention materials to their respective patients and providers.

This study is an open label non-blinded trial. Blinding is not required for this study as the intervention is delivered via mail and does not pose a risk for bias. Patients will be censored from the analysis at the time of disenrollment from the health plan that was in place at the time of trial enrollment, or at the time of death. Participants will be randomized to either the control arm or one of two intervention arms: the "provider only" arm and "patient/caregiver plus provider" arm. In the provider only arm, only the provider of the patient will receive provider level intervention materials; in the patient/caregiver plus provider education arm, both the patient/caregiver plus the provider will receive intervention materials. The provider will receive provider level intervention materials and the patient will receive patient level intervention materials. Providers and patients/caregivers will receive applicable educational materials through a one-time mailing

at trial start. In instances where a patient has been prescribed more than one inappropriate medication, only one educational intervention will be mailed based on the following hierarchy: antipsychotics first, followed by sedative-hypnotics and then strong anticholinergics.

PROVIDER-LEVEL INTERVENTIONS (PROVIDER ONLY ARM)

- Letters to providers:
  - Will explain the project and the rationale for why deprescribing is recommended in AD/ADRD patients. Evidence about potential drug harms for specific medications and options for safer alternatives will also be provided.
- Materials that were sent to the patients will also be provided.
- Website with the provider-focused information contained in the letters to providers. (knowmymeds.org)

PATIENT-LEVEL INTERVENTIONS (PATIENTS/CAREGIVERS AND PROVIDER ARM)

- Letters to Patients/Caregivers:
  - Will include information on potentially inappropriate medications and drug-specific brochures containing information about the use of certain medications that may be problematic.
  - The materials will also highlight the following recommendations:
    - Bring the enclosed materials to your next appointment with your doctor and ask him or her to review your list of medications to make sure you should continue taking all your medicines.
    - Do not stop taking a prescribed medicine unless your doctor says it is okay. Suddenly stopping a medicine can make symptoms worse or cause other problems.
- Pocket card with suggested questions to ask provider
- Website with the patient-focused information contained in the letters to patients. (knowmymeds.org)

PROVIDER-LEVEL INTERVENTIONS (PATIENTS/CAREGIVERS AND PROVIDER ARM)

- As described above in “PROVIDER ONLY ARM” intervention

Patients randomized to the control arm will receive usual care. No mailing will be sent to the patient or their physician.

Study specific website. Relevant to the intervention arms of the trial, a dedicated D-PRESCRIBE-AD study specific website will be available for patients, caregivers, and healthcare providers. The website will provide online access to all mailed educational materials. The website will provide a feedback form for patients, caregivers, and healthcare providers to anonymously provide feedback to the study team. The website url is knowmymeds.org.

Incoming phone calls from Humana participants will go to a toll-free study phone line that was provided by UMass Chan communications. Incoming phone calls from HealthCore participants will go to a toll free study line set up at Health Core. HealthCore research team will track calls received and send summary data to the UMass Chan study team with counts only; no identifiable information about members will be sent to UMass Chan. Only research team members will have the access code to retrieve voicemails on the UMass Chan study phone line. Details from the voicemail that are necessary to return the call will be recorded in REDCap (date of incoming call, participant name, reason for call). Study team members will return calls to participants within two business days (see attached phone script outlining guided responses for various potential topics). Clinical study team members who will be returning calls remotely will use the Doximity app to protect their personal contact information. Doximity is a HIPPA compliant app used by clinicians which masks the caller's contact information and replaces it with the phone number of your choosing. All returning phone calls to participants from UMass Chanwill appear as if they are coming directly from the study phone line 800 number so as to be recognizable to participants. Additional information about the security of Doximity app is provided below.

Security Operations

Doximity's team of security professionals ensure that our platforms and data are always protected by being SOC 2 Type 2 and HIPAA/HITECH certified. We conduct a variety of recurring security processes such as risk assessments, penetration testing (using internal testers and external firms), and white-box testing (with security researchers and security professionals).

HIPAA Compliance

Doximity's platform allows healthcare professionals to securely communicate while maintaining compliance with the Health Insurance Portability and Accountability Act of 1996 (HIPAA), as amended by the Health Information Technology for Economic and Clinical Health Act (HITECH). All Doximity employees and contractors who work on our systems that facilitate healthcare communications are required to complete ongoing HIPAA and security training.

Privacy

The privacy of our members and their work is paramount. Doximity does not sell or release the personal contact information of our members, including email addresses and phone numbers. Patient phone numbers are only used to connect our members to their patients through Dialer and are not used for any other commercial purposes. Voice and video calls made via Doximity are not monitored or recorded.

Any patient information that you provide to us in connection with your use of a secure communication tool that we make available through the Service, including Doximity Dialer, ("Patient Information"), will be used solely to provide the associated services to you and your patient. We do not monitor communication content and we do not record audio or video calls. We do not sell Patient Information.

12. DATA AND SPECIMEN BANKING*

N/A

13. Data Analysis and Management*

Results from the distributed cohort identification query are reviewed locally at each health plan site and securely returned to HPHCI and UMass Chan for analysis and reporting. Because querying is conducted in this distributed approach, it allows health plans to maintain physical and operational control of their data. Therefore, HPHCI and UMass Chan do not retain any protected health information (PHI) for this study.

Query results returned to HPHCI are retained onsite for a minimum of six years after the close of the study, followed by six years at an offsite storage facility. For file security, all desktops and laptops run encryption software from Credant Technologies, Inc. to prevent accidental loss or theft of data on computers or removable media from being usable. Network file storage is on a password protected server. Remote access to the Harvard Pilgrim network is available on Harvard Pilgrim laptops using the VPN software.

All health plans for this study are participants of the FDA Sentinel project and will use their approved local implementation of the Sentinel Common Data Model for querying. As participants in the Sentinel project, all health plans must undergo a rigorous data management and quality assurance process before their data is approved for use in querying. The frequency of each sites' quality assurance approval process depends on their specific contract with Sentinel, but occurs at a minimum on an annual basis. In addition to quality assurance of data elements, HPHCI adopts standard SAS programming quality assurance and quality control processes used by the Sentinel System to check SAS programs and deliverables.

The Principal Investigator and research team will comply with the University of Massachusetts Institutional Review Board requirements for defining, collecting and reporting any unanticipated problems, adverse events, or serious adverse events during the conduct of research.

Investigators will work with NIA to convene a Data Safety and Monitoring Board to oversee the human subjects' safety and adverse event reporting for this trial.

Sample Size and Power. Our target sample size for the first trial is 14,442 patients, 4814 patients in each of the three study arms. The calculations below employ 80% power, overall Type I error rate of .05 with a Bonferroni correction for 3 pairwise comparisons of study arms (.05/3=.0167), and 2-sided hypothesis testing. Based on our prior analyses,[26,27] we anticipate death or health plan disenrollment in 9.9% of sampled patients within 3 months of the intervention (receipt of the letter), with the remaining 90.1% contributing data in the 6-month interval of interest (days 91-270 post-intervention) – that is, we anticipate a per-arm sample size of 4814 × .901 = 4337.

For analyses of the primary outcome, absence of dispensing of targeted inappropriate prescription classes in days 91-270, we anticipate censoring in this interval for 13.5% of participants based on prior data. To make maximal use of observed data, we will use survival analysis to model time until an inappropriate prescription (a "failure") in days 91-270. Detectable pairwise between-arm differences (e.g., between usual care and an intervention arm) are presented in Table 2 below for a range of possible percentages for "failure" = inappropriate prescribing of the targeted drug.

Table 2. Detectable pairwise between-arm differences in hazard of inappropriate prescription classes in days 91-270

**Percent with inappropriate prescribing of targeted drug ("failure"), Study Arm 1**	**Detectable hazard ratio for inappropriate prescribing, Arm 2 versus Arm 1**
40	.8860
50	.8979
60	.9067
70	.9136
75	.9165
80	.9192
85	.9216
90	.9239
95	.9260
99	.9267

For the range of "failure" percentages examined here, which reflect those seen in Martin et al,[3] detectable hazard ratios range from 0.89 to 0.93. For example, if 75% of participants randomized to Arm 1 are observed to have a "failure" (prescription for a targeted inappropriate medication) by day 270, the detectable hazard ratio for an inappropriate prescription for Arm 2 versus Arm 1 is 0.9165, a 8.35% reduction in risk; the corresponding detectable "failure" probability for Arm 2 = 0.7193, a difference smaller – i.e., more precise – than that seen in Martin et al.[3] Calculations for the secondary outcome of ≥50% reduction in dose are parallel.

For additional secondary outcomes, such as per-patient number of hospitalizations or ED visits, based upon prior data (mean of 0.35 hospitalizations per 6-month period and 0.4 ED visits per 6-month period), we will be able to detect rate ratios of 0.8856 and 0.8927, respectively (corresponding to intervention-related reductions of 11.4% and 10.7%), accounting for censoring due to death or disenrollment. For between-arm differences in mortality, assuming usual care 6-month mortality of 6.3% – likely an underestimate given a lag in ascertainment – and 7.6% censoring due to disenrollment based on information provided by the participating health plans, the detectable hazard ratio is 0.7356 corresponding to per-arm survival percentages of 93.7% versus 95.33% (absolute difference of 1.63%).

Intention-to-Treat (ITT) Analysis Population (all randomized participants):

All analyses will be intention to treat. We do not expect differential loss to follow up between the three arms of the study. For the time to event analysis patients will be censored from the analyses

at the time of death, disenrollment from the health plan, loss of medical or pharmacy coverage, or change in eligibility for research based on health plan membership.

We will construct a detailed consort diagram showing the number of patients randomized to the three arms, the number of patients lost to follow up, excluded from analyses and the number of subjects included in the primary and secondary analysis.

In descriptive analyses, treatment arms will be compared regarding key patient characteristics, including age, gender, and renewal of inappropriate prescriptions in days 1-90, using percentages for categorical characteristics and means (standard deviations) or medians (interquartile range) for continuous characteristics, depending on the observed distributions. Analyses of study outcomes will employ two-sided hypothesis testing and an overall Type I error of 0.05, applying a Bonferroni correction to accommodate three pairwise comparisons of the three study arms. Covariates of *a priori* interest include patient age, gender, and renewal of inappropriate prescriptions in the blackout period. In addition, we will adjust for characteristics that are found to predict study outcomes, in order to increase precision for the comparison of study arms,[2] as well as for characteristics found to be related to censoring or other missing data, in order to reduce possible nonresponse bias. All analyses will be intention-to-treat. <u>Sex as a biological variable will be factored into all analyses</u>.

Statistical Analysis of Primary Outcome:

1. The primary outcome will be defined as absence of any dispensing of the selected inappropriate prescription class from day 91 to day 270 following the day of mailing. Educational interventions will be targeted towards one specific drug class so participants who switch within one class (e.g., clonazepam to lorazepam) will not be considered as having met primary outcome**. The intervention will only target one potentially inappropriate medication class for patients who are on more than one class of potentially inappropriate medication**.
2. **Timing of Ascertainment**: The timing of ascertainment is over a 6-month period beginning 3 months after mailing/intervention – i.e., we will assess evidence of a dispensing in days 91 through 270 after the date of mailing.
3. **Method of Aggregation: Hazard ratio**. The data on primary outcome will be measured as the relative hazard of time to dispensing of any new incident inappropriate prescription of their initial drug class in the intervention vs control group. The index date for the survival analysis will be Day 91 for the trial. We chose the hazard ratio as method of aggregation as this allows the statistical analysis to account for censoring due to death or disenrollment.
4. Any prescriptions dispensed during the **blackout period** will not be counted towards measurement of the primary outcome but may affect subsequent dispensing. In covariate-adjusted survival analyses, we will adjust for whether any prescription for the same inappropriate medication class was dispensed during the blackout period and the duration of such dispensing because dispensing during the blackout period is an important factor which may affect the primary outcome. A blackout period was needed to allow time for the

mailing and receipt of the intervention after randomization, and the opportunity to set up the appointment with or contact their provider to discuss the use of the potentially inappropriate medication. Alternatively, we may also consider stratification of survival analyses by prescriptions dispensed during the blackout period.

For the primary outcome (i.e., any post-intervention discontinuation of inappropriate prescribing during the 6-month period beginning 3 months after the mailing), as a first step, we will calculate crude arm-specific percentages, as well as Kaplan-Meier curves and log-rank testing. Covariate-adjusted comparisons of arms will be estimated using marginal Cox proportional hazards modeling. Comparing active intervention arms to usual care, we hypothesize a hazard ratio of less than 1, indicating lower risk of an inappropriate prescription in days 91-270 in the active intervention arms. The index date for the survival analysis will be day 91. We chose the hazard ratio as method of aggregation as this allows the statistical analysis to account for censoring due to death or disenrollment. To account for mortality, anticipated to be approximately 6%, we also will conduct competing risks analyses as well as cause-specific hazards modeling.

Analysis of the Secondary Endpoint(s):

Secondary outcomes. These will also be assessed specific to the 6-month observation period (days 91-270 following mailing/intervention) based on health plan claims data including:

a) Any dose reduction of each of the selected inappropriate medications, assessed at the participant level using health claims data (outpatient dispensings).
b) Decrease in the proportion of patients with prevalence of polypharmacy (defined as >5 active prescriptions for different agents).
c) Decline in the rates of emergency room visits; rates of hospitalizations; rates of non-acute institutional stays (e.g., skilled nursing facilities); overall health care utilization (number of outpatient visits, days hospitalized, emergency department visits, and non- acute institutional days).
d) Decline in-hospital all-cause mortality
e) Switching within drug class

We will use administrative claims data to identify encounters of interest (ED visits, hospitalizations, non-acute institutional stays, outpatient visits) and only assess oral formulations for medications.

Measurement of Dose Reduction. We will consider dose reduction for each selected drug as being a $\geq$ 50% decrease in the mean daily dose comparing the 6 months immediately prior to the randomization with the 6-month study window period. (day 91-day 270). We will measure average daily dose using dates of prescription dispensing, duration of prescription dispensing and strength of the prescription. We will measure the dose reduction as a dichotomous variable defined as the proportion of patients who achieved a more than 50% dose reduction in the daily dose during the study window period (day 91 to day 270) compared to the 6-month period prior to randomization. Measurement of dose reduction over 6 month follow-up requires participants to complete follow-up through end of observation period (day 270). Censored participants – that is, those who have less than 6 months in days 91-270 – will be excluded for the analysis of dose reduction. Analyses will

adjust for correlates of missing data. Analyses for post-intervention polypharmacy prevalence will be analogous. We will identify participants with evidence of dispensings of ≥5 oral medications over the respective 6-month periods [during the study window period (day 91 to day 270) compared to the 6-month period prior to mailing]. AD medications and the three potentially inappropriate medication classes will contribute to measure of polypharmacy. Injectables and topical or ocular medications will not be counted as evidence of polypharmacy.
A combination drug will be considered a single medication for the purpose of this analysis. Additional analyses will examine within-patient change in number of inappropriate medications, where the maximum possible decrease equals the pre-intervention number of inappropriate medications. We will accommodate this between-patient heterogeneity as follows: within-patient changes will be ranked separately by pre-intervention number of inappropriate medications, ranks will be transformed using normal scores to obtain comparable distributions across these strata, and treatment arms will be compared regarding transformed ranks[34] using analysis of covariance. In analyses of other secondary outcomes, count outcomes such as per-patient number of emergency department (ED) visits will be analyzed using Poisson or negative binomial regression, accounting for "excess" zeros if warranted based on observed distributions.[35]

Among study subjects who discontinue the targeted medication, we will determine if another agent within the targeted class has been dispensed over the period of observation (day 91-270).
For sedative/hypnotics: dispensing of a new generic agent within the class of sedative/hypnotics.
For antipsychotics: dispensing of a new generic agent within the class of antipsychotics.
Analyses will be analogous to those for dose reduction.

Mortality will be analyzed using survival analyses, including Kaplan-Meier curves, log-rank testing, and Cox proportional hazards (PH) modeling, accounting for censoring due to disenrollment

Baseline Descriptive Statistics

Demographics and baseline characteristics. Baseline and demographic characteristics will be based on claims data at the time of randomization. Frequency distribution and summary statistics will be presented by three intervention groups. Key demographics to be summarized include age in 5-year categories, sex, ethnicity, geographic region, Combined Comorbidity score, health care utilization indices and current use of inappropriate prescribing drugs. Categorical variables will be presented as frequencies and continuous variables as mean and SD. We will not use inferential statistics at baseline.

Sub-Group Analyses

We will conduct analyses stratified by sex and we do not anticipate sex related differences in within- group correlations (ICC).

Tabulation of Individual Participant Data

No individual participant data will be listed by measure.

Exploratory Analyses

Analyses parallel to those conducted for the primary outcome will be conducted by targeted inappropriate medication class**.**

14. Provisions to Monitor the Data to Ensure the Safety of Subjects*

While not anticipated, monitoring for any study safety will be overseen by the PI Jerry Gurwitz, MD, and the health plan site PIs as well as the site PI at Harvard Pilgrim HealthCare Institute. The project has no more than minimal risk because of the nature of the study activities. We will discuss any study safety issues during our regular team meetings and research team communications.

Data Safety Monitoring Board (DSMB). Though the proposed study is believed to pose no more than minimal risk to participants, the proposed study will involve multiple sites, thus a data and safety monitoring board/committee is deemed necessary. The DSMB will review any adverse events that are identified.

The identification and confirmation of inappropriate prescribing and other clinical outcomes will be via electronic health plan data pulls up to 15 months after enrollment in the study and the implementation of the intervention. Because the collection of this information is likely to be several months after occurrence, the information will not be actionable.

The DSMB will meet approximately every six months during the four years of the project to discuss study progress and procedures and the findings of interim analyses. Prior to study initiation, the DSMB will meet with the Principal Investigator and other key investigators to review the study protocol. Specific attention will be focused on the main study outcomes and their clear definition, the analysis plan, procedures for recording and reporting serious adverse events, the monitoring protocol, and responsibilities of the group. At the initial meeting, the DSMB may recommend modifications or request clarifications of the protocol. Also, it will formulate operating procedures for the group including: meeting schedules; expectations for reporting prior to each meeting; protocol-directed study stopping procedures; and interim data releases that will be allowed to the Principal Investigator.

See attached protocol and DSMB charter as approved by the DSMB.

Note: Regardless of what is written in the Manual of Procedures regarding reporting timelines to the DSMB, the study team will meet the minimum reporting requirements as outlined in HRP-103.

The NIA, the study funder, has appointed the following members of the DSMB:

Chiang-Hua Chang, PhD, MS
Research Assistant Professor, Geriatric and Palliative Medicine
University of Michigan

Laura C. Hanson, MD, MPH - chair
Professor, Geriatric Medicine
Medical Director, UNC Palliative Care Program
University of North Carolina, Chapel Hill

Michael Steinman, MD- Chair
Professor of Medicine
School of Medicine
University of California, San Francisco, CA

15. Withdrawal of Subjects Without Their Consent*

N/A

16. Risks to Subjects*

The proposed study poses low risk to participants. The risks that do exist fall into two categories: (a) risks associated with potential loss of confidentiality; and (b) risks associated with the research content area. We address each in turn below.

Potential Risks

Risks Associated with Potential Loss of Confidentiality. There is a slight risk that research records (data collection forms, electronic data) might be obtained by persons not authorized to do so. There is a slight risk that research data files might be compromised and obtained or viewed by unauthorized persons. Our procedures for protecting against such risks are described below.

Risks Associated with the Research Content Area. We recognize that the content of the materials may be emotionally sensitive (related to health conditions and healthcare practices of providers). Our procedures for protecting against such risks are described below.

Protections Against Risks

Minimizing Risks. The training and monitoring of all study staff performance in accordance with an IRB-approved study plan will be the responsibility of the Principal Investigator. All efforts will be made to minimize risks and participant inconvenience.

Recruitment. The recruitment methods will be reviewed and approved by the Institutional Review Board to ensure the protection of human subjects.

Risks associated with potential loss of confidentiality. The organizations proposing this study have systems, oversight, experienced personnel, and organizational cultures that support the appropriate use, access, and storage of confidential information. All persons collecting or handling data will be trained in human subjects' procedures, confidentiality, and privacy protection. All investigators and project staff are required to receive and complete IRB and HIPAA training.

Data for all participants will be kept strictly confidential. All hard copies of research files will be kept in locked file cabinets or a locked file room. Participants will be assigned a numerical code (Study ID) for identification in the files. Individual identifier information will be removed from study data files as soon as possible in the data processing steps. All computerized data will be kept on secured computers or networks. These data will be accessible only to research staff using confidential usernames and passwords. Statistical analyses will be performed using only limited datasets and only de-identified data will be reported. All data will be used for research purposes only; published data will not contain any individual identifiers.

All patient-level electronic data will be maintained by the health plans which have routine access to these data. Investigators who prepare reports, presentations, and publications based on this study will never have had access to identifiers of the complete study population. Investigators outside of the health plans will never have had access to any identifiers and will only receive

deidentified data and results. UMass Chan will execute Data Use Agreements with both health plans in order to receive the deidentified data.

HIPAA Authorization

Electronic Data. Electronic data from the administrative health plan systems will only be collected with the appropriate HIPAA Waiver as approved by the IRB. Electronic data will be collected for the purposes of: (1) recruitment for the clinical trial and to contact eligible patients with the intervention educational materials; and (2) outcomes assessment for those enrolled in the Clinical Trial. See section #27. Provisions to protect the privacy interests of subjects for more detail on the request for a HIPAA Waiver.

Content of Intervention Materials. All the materials for the study will be developed in collaboration with advisors and stakeholders and with feedback obtained through interviews with patients, caregivers, and providers to attempt to induce as little emotional distress as possible. Materials have been submitted to the IRB for review and approval for use with study participants in this study.

Vulnerable Subjects. See section 18 for protections related to vulnerable subjects.

17. POTENTIAL DIRECT BENEFITS TO SUBJECTS*

It is uncertain whether individual participants will directly benefit from participation. Some participants may learn something new about their health condition and/or treatment. Some participants may become motivated to specifically discuss medication management questions with their providers. Subjects may enjoy participating and may feel that doing so contributes to scientific knowledge in general.

18. VULNERABLE POPULATIONS*

See Section #6 on inclusion and exclusions. We will not enroll infants, children and teenagers, neonates of uncertain viability, or prisoners.

It is possible that health plan member data will be analyzed on (and only shared in the aggregate) pregnant women as this is not explicitly an exclusion criterion. No human subject will be exposed to any interventions that may harm a pregnant mother or fetus.

In the event of inclusion of pregnant women, the following three statements will hold true:

1. No inducements, monetary or otherwise, will be offered to terminate a pregnancy.
2. Individuals engaged in the research will have no part in any decisions as to the timing, method, or procedures used to terminate a pregnancy.
3. Individuals engaged in the research will have no part in determining the viability of a neonate.

We anticipate the inclusion of subjects with some cognitive impairment and dementia based on the inclusion criteria. However, this study poses no more than minimal risk to subjects, and we are requesting a waiver of consent. See also #30 Consent Process.

19. MULTI-SITE RESEARCH*

This study and all participating sites will comply with the NIH Policy on the Use of a Single Institutional Review Board for Multi-Site Research (NOT-OD-16-094). All identified participating sites have agreed to rely on the UMass Chan IRB. The PI and Project Manager at the UMass Chan will be responsible for managing all communications between participating sites and the IRB. The University of Massachusetts Medical School will maintain records of the authorization/reliance agreements and of the communication plan. All participating sites will, prior to initiating study activities, sign an authorization/reliance agreement that will clarify the roles and responsibilities of the IRB and participating sites.

The central project office will be located at **UMass Chan.** The Principal Investigator, Dr. Gurwitz, will oversee all project activities. The UMass Chan project manager, with support from the UMass Chan research assistant, will coordinate all meetings, conference calls, dissemination of materials, and tracking of task completion. As we have in previous multi-site studies, to maximize clear communication and efficient study management, we will utilize the following procedures: (1) a cross-site working group will be established for investigators and project managers; this group will hold regular conference calls coordinated by staff from the central project site who will develop and distribute agendas and action-oriented minutes; Dr. Gurwitz will participate in these calls; (2) detailed timelines will be developed and distributed for each aspect of the project; (3) tracking systems will monitor study progress.

20. COMMUNITY-BASED PARTICIPATORY RESEARCH*

N/A

21. SHARING OF RESEARCH RESULTS WITH SUBJECTS*

There are no specific plans to share results with study subjects; study procedures do not include any type of diagnostic testing. All results shared in published research will be in aggregate or summary format and will not include identifiable information about participants. Published results will be available to the greater community at large, including study subjects.

22. SETTING

Health Plan Activities

Health plan data queries. This project will employ the NIH Collaboratory Distributed Research Network infrastructure based at Harvard Pilgrim HealthCare Institute which uses the FDA Sentinel Initiative infrastructure. The FDA Sentinel Initiative, established in 2009, is a long-term public health surveillance program designed to create a national electronic system for monitoring the safety of FDA-regulated drugs and other medical products. The Sentinel Initiative includes a wide array of collaborating organizations, across the United States including health plans, which are referred to as Data Partners. For this analysis we will be using two Data Partners Humana and Health Core. The electronic data used in the process is accessed, maintained, and protected, as part of a "distributed system." In a distributed system, data remain in their existing secure environments, rather than being consolidated into a single database. Data Partners maintain physical and operational control over their electronic health data behind their institutional firewalls. Data Partners transform their data into the Sentinel Common Data Model on a regular

basis, execute standardized analytic queries distributed by the Sentinel Operations Center, which is based at Harvard Pilgrim HealthCare Institute (HPHCI), then share the output of queries, typically in summary form, with the Operations Center at HPHCI via a secure network portal. This system protects the privacy and confidentiality of individual-level health information and is preferred by participating health plans over a centralized data repository approach. **Both health plans will also be sharing deidentified data sets with UMass Chan medical school; Data Use agreements with both plans will be implemented in order to do so.**

23. Resources Available

All research personnel listed on this study will read the protocol and receive the appropriate supervision and possess the appropriate experience (both higher education and related work experience) needed to fulfill their roles and complete their responsibilities for this study. All investigators and project staff are required to receive and complete IRB and HIPAA training.

The Principal Investigator will oversee all personnel and all research activities conducted within this study.

The Principal Investigator will have responsibility for the overall conduct of the project at this study site. He will have primary oversight of all study personnel. He will participate in the design and the execution of the respective study analyses and will be responsible for the reporting of study results.

The Co-Investigators will participate in all aspects of the research and help to ensure the accomplishment of all study goals. They will participate in designing, developing, and implementing study procedures and materials. They will participate in project-related calls and meetings and help to develop deliverables and participate in manuscripts. The Co-Investigators will assist the Principal Investigator in research design and intervention development as well as analytic aspects of the study. The Co-Investigators include clinicians and researchers with varied expertise including: healthcare services research, qualitative and quantitative research design, health communication and health literacy, and biostatistics.

The Pharmacist Consultant will assist the Principal Investigator in research design and provide content expertise.

The Biostatistician will assist in the design and performance of analyses relevant to the project and will assist in the development of study deliverables.

The Project Manager will assist the Principal Investigator and the Co-Investigators in implementing all aspects of the project. Under the direction of Principal Investigator, the Project Manager will be responsible for day-to-day coordination and oversight of the project, including: developing timelines, work allocation, workflow plans, monitoring project progress and task completion, monitoring spending and effort allocation, and managing correspondence and administrative tasks. S/he will monitor/manage ethics and regulatory approvals (IRB, HIPAA/DUA). The Project Manager will attend and plan for all project-related meetings as needed. S/he will work under the direction of the Principal Investigator to assist with all study activities, preparing IRB submissions and reports, and developing study materials such as development of data collection instruments and intervention-related tools. S/he will be responsible for maintaining communications with all parties participating in the project. S/he will maintain project documentation and will assist in developing and filing required project

reports. The project manager may also obtain informed consent. Project Managers at UMass Chan Health Service Sciences all hold graduate level degrees and have vast experience working on healthcare services research projects.

The Research Assistant will work under the direction of Principal Investigator, Project Manager and co-investigators to assist with all study activities. S/he will assist the PI and other project staff in managing the administrative activities of the project. S/he will prepare materials for team meetings and will facilitate communication between all project staff through written correspondence, telephone, fax, and email. The research assistant may also obtain informed consent.

The Stakeholder Panel members (approximately 3 patients and/or caregivers, 2 health care providers, 2 health plan leaders) and the Expert Advisory Panel members (approximately 5 experts) will be engaged in an advisory capacity only. These members are neither considered study subjects nor study personnel, but rather an expertise resource for the study team. They will neither interact with subjects nor access private identifiable information about them.

All study personnel are required to undergo Human Subjects Training and hold a current CITI Human Subjects Training Certificate and will familiarize themselves with the study protocol and IRB documents.

24. Local Recruitment

N/A (see section #8 Study-Wide Recruitment Methods)

25. Local Number of Subjects

See section #7 Study Wide Number of Subjects.

26. Confidentiality

<u>Health Plan Activities</u>

Electronic Clinical and Administrative Patient Data

This project will employ the NIH Collaboratory Distributed Research Network infrastructure based at Harvard Pilgrim HealthCare Institute which uses the FDA Sentinel Initiative infrastructure. The FDA Sentinel Initiative, established in 2009, is a long-term public health surveillance program designed to create a national electronic system for monitoring the safety of FDA-regulated drugs and other medical products. We will be working with two health plans, Health Core and Humana which are part of the network. The Sentinel Initiative includes a wide array of collaborating organizations across the United States, including health plans, which are referred to as Data Partners. The electronic data used in the process is accessed, maintained, and protected, as part of a “distributed system.” In a distributed system, data remain in their existing secure environments, rather than being consolidated into a single database. Data Partners maintain physical and operational control over their electronic health data behind their institutional firewalls. Data Partners transform their data into the Sentinel Common Data Model on a regular basis, execute standardized analytic queries distributed by the Sentinel Operations Center, which is based at Harvard Pilgrim HealthCare Institute (HPHCI), then share the output of queries, typically in summary form, with the Operations Center at HPHCI via a secure network portal. This system protects the privacy and confidentiality of individual-level health information

and is preferred by participating health plans over a centralized data repository approach. **Both health plans will also be sharing deidentified data sets with UMass Chan medical school; Data Use agreements with both plans will be implemented in order to do so.**

Coordination and Security

A key benefit of the distributed approach is that it minimizes the need to share identifiable patient information. Data Partners are also able to review the results of the queries before sending them back to the Operations Center, which is based at Harvard Pilgrim HealthCare Institute.

All health plan source data files are stored behind a secure firewall on computers with virus software and will be accessed only by the programmers working on this study. Data transfer between Data Partners and the Operations Center, as well as UMass Chan Medical School, is done by means of a secure web-based file sharing system. The Operations Center complies with standards established by the Health Insurance Portability and Accountability Act of 1996 (HIPAA) and the Federal Information Security Management Act of 2002 (FISMA). The Operations Center information technology infrastructure is provided by Harvard Pilgrim HealthCare Institute and supported by Perot Systems. All computing resource access is managed in compliance with HIPAA Security standards.

<u>Data Storage and Processes</u>

Computerized Data Storage

All computerized data will be kept on secured computers or network servers, behind UMass Chan firewalls. These data will be accessible only to research staff with approved access, using confidential usernames and passwords.

Paper Data Storage

Any paper data will be kept in locked cabinets or a locked file room accessible only by research staff.

Data Processing

Analyses will be performed using only limited data sets and only de-identified data will be reported. All data will be used for research purposes only; published data will not contain any individual identifiers and will be reported in the aggregate.

27. Provisions to Protect the Privacy Interests of Subjects

<u>Health Plan Activities</u>

Health Plan Data Queries. The analysis of electronic health records by the health plans will be conducted under a HIPAA waiver. Data will only be available to the health plan entities which readily have access to those data; **<u>any data shared for research analyses will be deidentified</u>**. Identifiers will remain with the participating health plans.Data will remain behind secure

firewalls at the health plans and will not be accessed by any personnel who do not already have routine access as part of normal business operations.

We request a HIPAA waiver of authorization for the health plan related activities (see detailed information on the HIPAA Waiver Request form). We believe the study meets the following criteria to obtain a waiver of consent (followed by a *rationale*):

We believe the study meets the following criteria to obtain a waiver of HIPAA Authorization (followed by a rationale):

1. The use or disclosure of protected health information involves no more than a minimal risk to the privacy of individuals.
   Rationale: The release of individual PHI will be only to the health plan entities which readily have access to those data. The intervention is also entirely consistent with a quality improvement initiative that the health plans could initiate on their own.

2. There is an adequate plan to protect the identifiers from improper use and disclosure.
   Rationale: Identifiers will remain with the participating health plans; no disclosure of individual-level patient data will occur beyond the health plan of origin. Data will remain behind secure firewalls at the health plans and will not be accessed by any personnel who do not already have routine access as part of normal business operations.

3. There is an adequate plan to destroy the identifiers at the earliest opportunity consistent with conduct of the research unless there is a health or research justification for retaining the identifiers or such retention is otherwise required by law.
   Rationale: Identifiers, which will remain at the health plan of origin, will be destroyed as soon as all data are collected, verified, and analyzed.

4. There are adequate written assurances that the protected health information will not be reused or disclosed to any other person or entity, except as required by law, for authorized oversight of the research study, or for other research for which the use or disclosure of the protected health information would be permitted.
   Rationale: PHI will not be disclosed beyond the health plan of origin nor for use beyond the scope of the research aims of this study. The Sentinel System has established systems for data management and security.

5. The research could not practicably be conducted without the waiver or alteration.
   Rationale: There are several reasons why the research would be impractical without the waiver of authorization. First, contacting "control" and "provider only intervention" patients for authorization would be an intervention by itself and might affect the results of the study. Secondly, given the number of subjects to be included in the pilot and the trials, it would be impractical to collect authorization from the total study population included in the research.

6. The research could not practicably be conducted without access to and use of the protected health information.
   Rationale: The research could not practicably be conducted without access to and use of the PHI as some PHI is required to identify eligible patients (e.g., date of birth, date of

dispensing); PHI is further required to contact eligible patients with the intervention educational materials (name, address), and PHI is required to assess the outcomes (e.g., dates of dispensings of medications of interest).

7. Access to the protected health information is necessary.
   Rationale: As described above, access to the PHI is necessary to conduct the research.

Additionally, there are adequate written assurances that the protected health information will not be reused or disclosed to any other person or entity, except as required by law, for authorized oversight of the research study, or for other research for which the use or disclosure of the protected health information would be permitted. We will not disclose study data beyond the health plan of origin nor for use beyond the scope of the research aims of this study.

28. COMPENSATION FOR RESEARCH-RELATED INJURY

N/A; we do not anticipate any research-related injuries. We believe the research poses no more than minimal risk to subjects.

29. ECONOMIC BURDEN TO SUBJECTS

N/A

30. CONSENT PROCESS

We are requesting a waiver of consent from the IRB for the Randomized Clinical Trial for both patients and providers. We believe the study meets the following criteria to obtain a waiver of consent (followed by a rationale):

1. The research involves no more than minimal risk to the subjects.

   Rationale: The probability and magnitude of harm or discomfort anticipated in the research are not greater in and of themselves than those ordinarily encountered in daily life or during the performance of routine physical or psychological examinations or tests. The intervention is entirely consistent with a quality improvement initiative that the health plans could initiate on their own. The intervention only adds to the existing care of patients. There are no restrictions placed on the control group as a result of the trial.

2. The waiver or alteration will not adversely affect the rights and welfare of the subjects.

   Rationale: The waiver of consent will not impede on any rights or the welfare of subjects; the waiver will solely allow the research team to implement the educational intervention by mail which subjects may choose to entirely ignore.

3. The research could not practicably be carried out without the waiver or alteration.

   Rationale: There are several reasons why the research would be impractical without the waiver of consent. First, contacting “control” and “provider only intervention” patients for consent would be an intervention by itself and might affect the results of the study. Secondly, given the number of subjects to be included, it would be impractical to collect informed consent from the total study population included in the trial.

4. Whenever appropriate, the subjects will be provided with additional pertinent information after participation.

Rationale: If necessary, we will provide additional information to patients and providers, as deemed appropriate by the IRB.

5. The research does not involve non-viable neonates as subjects.

   Rationale: The research only includes living adult patients aged 50 years of age or older and/or their medical providers.

6. The research could not practicably be carried out without using Identifiable Private Information.

   Rationale: The research could not practicably be conducted without access to and use of the Identifiable Private Information as some Private Identifiable Information is required to identify eligible patients (e.g., date of birth, date of dispensing); Private Identifiable Information is further required to contact eligible patients and their providers with the intervention educational materials (name, address), and Private Identifiable Information is required to assess the outcomes (e.g., dates of dispensings of medications of interest).

31. PROCESS TO DOCUMENT CONSENT IN WRITING
N/A

32. DRUGS OR DEVICES
N/A; this research does not involve testing drugs or devices.
