# Supplementary material for: Developing a PRogram to Educate and Sensitize Caregivers to Reduce the Inappropriate Prescription Burden in the Elderly with Alzheimer’s Disease (D-PRESCRIBE-AD): Trial protocol and rationale of an open-label pragmatic, prospective randomized controlled trial
Source: PLoS One. 2024 Feb 12;19(2):e0297562. doi: 10.1371/journal.pone.0297562 (PMC10861034; doi:10.1371/journal.pone.0297562)
Supplement: S4 Appendix — (PDF) [file pone.0297562.s006.pdf]

Health Plan Logo

Date

Suggested Actions:
✓ Review enclosed information<br>✓ Discuss with your patient and make any warranted changes in therapy

Dear Dr. Smith,

This letter contains information that may assist you in caring for your patients. According to our records a patient of yours may be taking Lorazepam.

Sedative-hypnotic medications often have side effects including falls and fractures, dizziness, memory problems, and daytime fatigue. Experts recommend that **these potentially inappropriate medications are best avoided by older adults** in most circumstances.[1]

Alert that drug may be inappropriate for patient.

We have mailed a patient information sheet on sedative-hypnotic medications to the following patient (a copy is enclosed).

Name: **John Doe**

Date of Birth: **01/01/1945**

Medication: **Lorazepam**

Date Initiated: **01/01/2018**

Patient specific information.

Reference to enclosures.

We have enclosed an **algorithm** that may help you decide whether to reduce or discontinue this medication, and a **tapering guide** to help patients understand and track dose reductions. This, along with the patient information sheet, are available at knowmymeds.org.

We realize that our records may not reflect all clinically relevant information or may be incomplete. If you have questions or concerns about this letter, please contact us at [contact information].

Sincerely,

Chief Medical Officer
Health Plan

This effort is a collaboration between [Health Plan] and a National Institute on Aging-funded initiative to increase awareness in patients, caregivers, and clinicians about medications that may be unsafe in older adults with dementia.

[1]2019 American Geriatrics Society Beers Criteria® Update Expert Panel. American Geriatrics Society 2019 Updated AGS Beers Criteria® for Potentially Inappropriate Medication Use in Older Adults. J Am Geriatr Soc. 2019 Apr;67(4):674-694.
